# Insights into the Loblolly Pine Genome: Characterization of BAC and Fosmid Sequences

**DOI:** 10.1371/journal.pone.0072439

**Published:** 2013-09-04

**Authors:** Jill L. Wegrzyn, Brian Y. Lin, Jacob J. Zieve, William M. Dougherty, Pedro J. Martínez-García, Maxim Koriabine, Ann Holtz-Morris, Pieter deJong, Marc Crepeau, Charles H. Langley, Daniela Puiu, Steven L. Salzberg, David B. Neale, Kristian A. Stevens

**Affiliations:** 1 Department of Plant Sciences, University of California Davis, Davis, California, United States of America; 2 Department of Evolution and Ecology, University of California Davis, Davis, California, United States of America; 3 Children's Hospital Oakland Research Institute, Oakland, California, United States of America; 4 Center for Computational Biology, McKusick-Nathans Institute of Genetic Medicine, Johns Hopkins University, Baltimore, Maryland, United States of America; Universidad Miguel Hernández de Elche, Spain

## Abstract

Despite their prevalence and importance, the genome sequences of loblolly pine, Norway spruce, and white spruce, three ecologically and economically important conifer species, are just becoming available to the research community. Following the completion of these large assemblies, annotation efforts will be undertaken to characterize the reference sequences. Accurate annotation of these ancient genomes would be aided by a comprehensive repeat library; however, few studies have generated enough sequence to fully evaluate and catalog their non-genic content. In this paper, two sets of loblolly pine genomic sequence, 103 previously assembled BACs and 90,954 newly sequenced and assembled fosmid scaffolds, were analyzed. Together, this sequence represents 280 Mbp (roughly 1% of the loblolly pine genome) and one of the most comprehensive studies of repetitive elements and genes in a gymnosperm species. A combination of homology and *de novo* methodologies were applied to identify both conserved and novel repeats. Similarity analysis estimated a repetitive content of 27% that included both full and partial elements. When combined with the *de novo* investigation, the estimate increased to almost 86%. Over 60% of the repetitive sequence consists of full or partial LTR (long terminal repeat) retrotransposons. Through *de novo* approaches, 6,270 novel, full-length transposable element families and 9,415 sub-families were identified. Among those 6,270 families, 82% were annotated as single-copy. Several of the novel, high-copy families are described here, with the largest, *PtPiedmont*, comprising 133 full-length copies. In addition to repeats, analysis of the coding region reported 23 full-length eukaryotic orthologous proteins (KOGS) and another 29 novel or orthologous genes. These discoveries, along with other genomic resources, will be used to annotate conifer genomes and address long-standing questions about gymnosperm evolution.

## Introduction

Gymnosperms have undergone 300 million years of evolution since their divergence from the ancestors of modern angiosperms, and they possess enormously complex genomes in comparison [1]–[3]. Increased ploidy level and individual repeats in high copy number are common in angiosperms but are rarely seen in gymnosperms [4], [5]. While rapid progress has been made in characterizing the genomes of angiosperms, the same is not true for gymnosperms, in part due to an order of magnitude increase in their size and complexity. Conifers are by far the most important representatives of the gymnosperms, prevalent in a variety of ecosystems and representing 82% of terrestrial biomass [6]. Comparative studies have demonstrated that they are characterized by reduced coding region evolution, retrotransposon proliferation, highly diverged repetitive sequences, accumulation of noncoding regions, and extensive gene duplication [3], [4], [7], [8]. Until recently, sequencing conifers has been nearly impossible due to the assembly complexity and sheer magnitude of sequence (*Taxodium distichum*: 9.7 Gbp, *Picea abies*: 19.6 Gbp, *Pinus banksiana*: 22.3 Gbp) [9]. Recent advances in the cost and utility of second generation, high-throughput sequencing technologies have made it possible for ten conifer reference genomes to be assembled (http://www.pinegenome.org/pinerefseq). Together, these will aid breeding efforts and illuminate mechanisms behind adaptive diversity for managing forest populations.

The largest genera in the order Coniferales, *Pinus,* comprises over 100 species and accounts for over 40% of global forest plantations [10]. Loblolly pine, a relatively fast-growing and economically important representative of the conifers, is a forest tree species native to the Southeastern United States. Traditional commercial markets for loblolly pine have included lumber, pulp, and paper, but more recently, it has become a major bioenergy feedstock in lignocellulosic ethanol production [11]. Recent estimates [9] place the size of the loblolly pine genome between 21 and 24 Gbp. In the context of completed genome projects, this is approximately seven to eight times larger than the human genome, and about four times the size of the largest angiosperm for which we have a reference genome, *Hordeum vulgare* (barley) [12]. Large-scale EST discovery projects have generated significant transcriptomic resources in the absence of a reference genome, including 328,662 ESTs, 17,379 UniGenes, and over 200,000 contigs from Roche/454 sequencing [13], [14]. The current genomic resources for loblolly pine include 103 Bacterial artificial chromosomes (BACs) totaling 10 Mb. BACs are the most popular genomic DNA construct and have been used extensively for genome characterization and in hierarchical sequencing projects like the human genome [15], [16]. BAC libraries offer a source of large genomic inserts (up to 200 Kbp) that facilitate studies of genomic content without a reference sequence [17]. They have been used in genomic studies of other gymnosperms, including *Pinus pinaster* [18], *Picea glauca* [19] and *Taxodium distichum* [20]. In addition to the BAC resource, we have generated just over 90,000 fosmid sequences totaling 265 Mb. Fosmids have been used to characterize several plant genomes, including sugar beet [21], Maire yew [22], cucumber [23], and strawberry [24]. Fosmids are less expensive to generate than BACs and have less cloning bias; their insert size can also be more narrowly controlled [25]. Like BACs, they are an ideal resource for preliminary genome characterization and for improving whole-genome shotgun assemblies.

While several categories of transposable elements (TEs) exist in plant genomes, the Class I elements known as long terminal repeat (LTR) retrotransposons are most frequently associated with genome size variation [26]. Studies across several plant species have noted that polyploidization and rapid proliferation of TEs can each lead to genome expansion [27]. Polyploid angiosperms such as *Oryza sativa, Vitis vinifera,* and *Hordeum vulgare* have repetitive content estimates of 35%, 41.4%, and 84% respectively [12], [28]–[30]. The diploid genome of a wild species of rice, *Oryza australiensis*, with a repetitive content estimate of 76%, has doubled in size over the last three million years through the expansion of a select few retrotransposon families [31]. Phylogenetic inference of gymnosperm lineages provides strong evidence for a recent, large increase in genome size in pines without associated acceleration of phenotypic diversity, suggesting periods of retrotransposon expansion [32]. The repetitive content of conifers *Picea glauca, Pinus taeda,* and *Taxodium distichum* have been estimated, through analysis of BAC sequences, to be anywhere from 20% to 90% [7], [19], [20]. Characterization of *Taxus mairei* fosmid sequences yielded a repetitive content estimate of 20.8%, excluding tandem repeats [33]. As in many plant genomes, an abundance of TEs has been noted in conifers, specifically those of the Copia and Gypsy families [34]. A handful of these LTRs have been characterized, and their distributions suggest that conifer genomes have very diverse and abundant repetitive sequences [3], [7], [35]. Most of these elements, however, remain uncharacterized.

In this study, we present a comprehensive analysis of repeats in both the existing BACs (11 sequences provided by [7] and 92 sequences available in Genbank [35] and an extensive novel fosmid resource. The fosmid set is the largest genomic resource generated to date for a gymnosperm, and consists of 90,954 scaffolds assembled from fosmid pools. Interspersed and tandem repeats were detected using both homology-based searches, which identify repetitive elements based on similarity to existing elements in a repeat library, and *de novo* searches, which scan for repeats without prior knowledge of repeat element sequence. Of particular interest are previously undiscovered repetitive element families, which were analyzed and annotated. In addition, a combination of orthologous proteins and *ab initio* gene prediction approaches were employed to characterize the gene space. These efforts generated a more comprehensive view of repetitive content for loblolly pine, as well as a bioinformatic framework for full genome annotation.

## Materials and Methods

### Fosmid DNA Prep, Library Construction, Sequencing

Genomic DNA from *Pinus taeda* genotype 20–1010 diploid needle tissue was isolated and extracted using a modified version of the method [36]. The extracted genomic DNA was then sheared to an average size of approximately 40 Kbp and size-purified by pulsed-field electrophoresis. This was followed by ligation to excess vector, pFosTH, (dephosphorylated ends) and vector packaging (extracts from mcrA, B, C strains) to create a particle library. The particle library was then titered, portioned, and converted into *E. coli* colonies. This procedure resulted in 11 pools of approximately 580+/−10% *E. coli* colonies, each colony containing a single fosmid.

The 11 harvested bacterial colony pools were then amplified *in vivo*. Fosmid DNA was subsequently purified and digested with the homing endonuclease PI-SceI, which has a 35 bp recognition site. With the isolated DNAs quantified by PicoGreen, the purified insert DNAs were portioned out to create an equimolar super-pool of all 11 component fosmid pools, as well as a set of three nested equimolar super-pools of 8, 4, and 2 small fosmid pools. The two smallest pools were not considered further in this analysis.

The fosmid DNA was processed into paired-end Illumina libraries for subsequent second generation sequencing. The largest super pool (of 11 pools) was converted to a long insert jumping library, and the smaller nested super pool (of 8 pools) was converted to a short insert fragment library. Standard Illumina reagents and protocols were used for library construction. Large fragments were generated with a HydroShear and small (sub-kilobase) fragments were generated with a Diagenode Bioruptor.

Paired-end libraries were subsequently sequenced on an Illumina GA2x [SCS version 2.9.35, RTA version 1.9.35]. Approximately 45 million 125 bp paired-end reads were obtained from the fragment library while 27 million 125 bp paired-end reads were obtained from the jumping library. Subsequent alignment of the reads to the *E. coli* and vector genomes was used to determine median insert size. Ungapped single seed alignment was done using Illumina's CASAVA pipeline version 1.7.0. GERALD reported a median insert size of 3,300 bp for the jumping library with a coefficient of variation of approximately 5%. The shorter fragment library had a median insert size of 258 bp with a coefficient of variation of approximately 4%. The reported rates of the non-canonical paired-end alignment orientations were also consistent with high quality libraries.

### Fosmid Assembly

Prior to assembly, the reads were processed with multiple methods designed to improve the overall assembly. The reads were quatity (Q20) and adapter trimmed using the “fastq-mcf” program, part of the “eautils” [37]. Following the trimming, the reads were aligned using BWA to a contaminant database composed of pFosTH, Ecoli, chloroplast and mitochondrion sequences [38]. The mate pairs that had one or both reads aligned to this database were discarded. The fosmid pool sequences were assembled using SOAPdenovo v1.05 [39] with a k-mer size of 63. Four iterations of GapCloser (v1.12) with a SOAPdenovo rescaffolding step in the middle were used for gap closing and assembly improvement. All scaffolds of lengths >200 bp were submitted to Genbank (WGS: APFE01000000).

### BAC Sequencing and Assembly

Two sets of loblolly pine BAC sequences derived from the Mississippi Genome Exploration Laboratory (MGEL) and Clemson University Genomics Institute (CUGI) libraries [35], [36] were analyzed here. Ten BAC clones (Genbank Accession Nos. GU477256-GU477266) were individually Sanger sequenced and assembled with Arachne [7]. The average read depth of each BAC assembly ranged from 6x to 16x. Nine of the BAC assemblies were resolved into a single scaffold and the last resolved into two unoriented contigs. An additional set of 92 BAC clones (Genbank Accession Nos. AC241263-AC241362) from the same libraries were sequenced with 454/Roche Pyrosequencing with an average read depth of 7x.

To eliminate potential redundancy between the independently derived fosmid and BAC datasets, three fosmid scaffolds identified as exact matches (99% identity and full coverage) against the longer BAC sequences were removed. In addition, 85,675 assembled fosmid contigs with lengths <201 bp were removed from the final set.

### Repeat Identification

**Figure 1:**
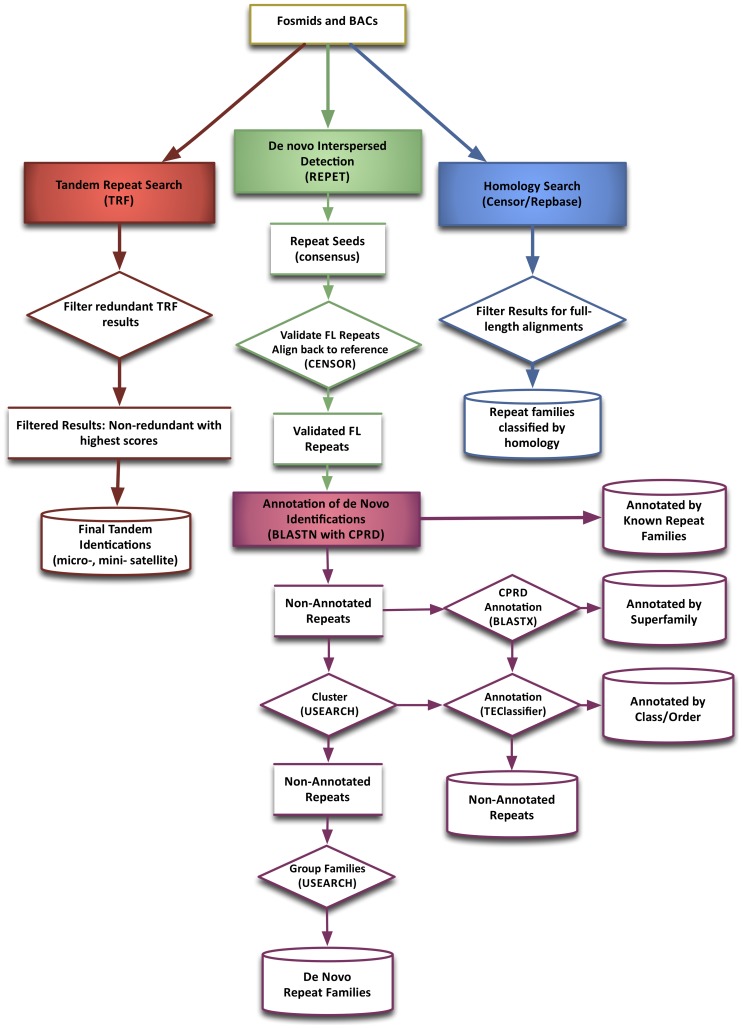
Repeat detection methodology. BAC sequences (103) and fosmid sequences (90,954) were analyzed for tandem repeats (TRF), interspersed repeats by homology (CENSOR against CPRD), and interspersed repeats via *de novo* methods (REPET). Details of the annotation process are also shown.

A combined methodology of homology-based and *de novo* approaches was used to identify the tandem and interspersed repetitive content in the BAC and fosmid sequences (Figure 1).

### Tandem Repeat Identification

Tandem Repeat Finder (TRF) v4.04 [40] was run with default parameters to identify tandem repeats in the BAC and fosmid sequence sets. Repeat units, or periods, of sizes 2 to 500 bp were considered. Mononucleotides (period size of 1) were not included due to the high likelihood of repeat collapse in the assembly process. To accurately assess the coverage of tandem repeats, we filtered overlaps by discriminating against hits with low TRF-based alignment scores. For comparison, these methods were applied to the reference genomes of *Arabidopsis thaliana* v1.6.7, *Vitis vinifera* v1.4.5, *Cucumis sativus* v1.2.2, and *Populus trichocarpa* v2.1.0, available through Phytozome [41]. In addition, BAC sequences (Table S1) from *Picea glauca* (white spruce) and *Taxus mairei* (Maire yew) were analyzed (Genbank Accession Nos: 262263013, 221149199; 327410373- 327412295). NCBI-BLASTN v2.2.26 was run with default parameters to verify whether particular satellites were specific to *Pinus taeda*.

### Interspersed Repeat Identification (Homology)

Censor v4.2.27 [42] was used to annotate repeats through homology. Censor utilizes NCBI-BLASTN (by default) to align queries against a database of curated repetitive elements. For this analysis, a custom plant repeat database (CPRD) was constructed using a subset of Repbase v17.07. This database is composed of plant repeats (plnrep, grasrep, mcotrep, dcotrep, oryrep and athrep) as well as five TEs available in the literature and previously classified in forest trees: TPE1 from *Pinus elliottii* [43], PpRT1 from *Pinus pinaster* [44], *Gymny* from *Pinus taeda* [3], PtIFG7 from *Pinus taeda* [7], and Corky from *Quercus suber* [45]. Censor makes available three sensitivity modes (normal, rough and sensitive) along with varying BLAST parameters to balance sensitivity and speed. The normal mode (blastall -p blastn -b 10000000 -Y 1000000 -e 0.00002 -a 64 -r 3 -q -4 -y 80 -X 130 -Z 150 -W 10 -J T -m 8 -i QU -d DB -F ‘m D’) was selected, as it yielded the same or improved alignments when compared against the sensitive mode for our datasets. Runs were performed in both redundant (a region may be annotated more than once) and non-redundant (the annotation with the highest alignment score is chosen) modes to provide a basis for species comparisons. Full-length hits and repeats that belonged to previously characterized families were identified using a modified version of the 80-80-80 standard [46]. This dictates that alignments should be >80 bp in length with at least 80% identity, and should cover 80% of the reference sequence. In this instance, percent identity was substituted with Censor's *sim* metric, and is defined as: (match_count/( alignment_length – query_gap_length – subject_gap_length + gap_count).

### De Novo Interspersed Repeat Identification

*De novo* repeat identification focuses on the discovery of repetitive sequence through self-alignment and/or pattern matches for structural features. To implement *de novo* repeat identification, the BACs (103 sequences) and fosmids (90,954 sequences) were run through the REPET TEdenovo pipeline (v2.0) with default settings [47]. This software wraps several open-source tools to identify, characterize, and derive a consensus sequence for each element. The first stage is self-alignment (all vs all) with NCBI BLAST 2.2.25 to detect high-scoring segment pairs (HSPs) [48]. This is followed by three separate rounds of clustering with three independent algorithms for repeat identification, including Grouper [48], Recon [49] and PILER [50]. Up to three clusters for each element are aligned with the multiple sequence aligner, Map [51], to derive a consensus. A structural search is run simultaneously with LTRharvest to identify highly diverged LTR retrotransposons [52]. The results from this secondary search are clustered with BLASTCLUST to group candidate sequences [53]. The results from both pipelines are combined, and subsequently filtered to reduce the redundancy generated from independently derived consensus sequences.

TEclassifier, a secondary REPET application, provides a first-pass annotation of the consensus sequences. This application delivers annotations by running BLASTER against four databases [47]: Repbase 17.07 (in both nucleotide and amino acid formats), a Pfam protein domain HMM database specifically formatted for REPET, a database of known genes from the host organism (for which we used 366 full-length *Pinus taeda* cDNAs and 945 *Pinus radiata* full-length cDNAs [54]), and a database of ribosomal DNA sequences from the host organism (for which we used 52 ribosomal *Pinus* sequences). Poly-A tails, tandem repeats, open reading frames, terminal repeats, and tRNAs are also are detected at this stage. After the database searches and filters, order-level annotations are provided according to the repeat classification system [46].

To contend with false positives and partial sequences, all sequences identified by REPET were treated as repetitive element *seeds*. In this context, seeds are *de novo* consensus sequences used as a library. The seeds from both sequence sets were combined and submitted to a non-redundant Censor search in normal sensitivity mode against the BAC and fosmid references. This enabled the full-length repeats to be mapped and aligned back to the reference. Alignment statistics and positions were recorded in a local MySQL database to facilitate downstream queries. All raw full-length hits were extracted from this database, observing the modified 80-80-80 standard described above.

### De Novo Interspersed Repeat Annotation

To effectively combine the homology and *de novo* search results, sequences corresponding to the full-length hits were extracted and compared against the homology database, CPRD. These alignments were manually classified and annotated into (Class: Order: Superfamily) based on the curated descriptions available from the database following a variation of the three-step classification procedure [46]. Combined with the 80-80-80 standard, BLASTN was used to further classify elements into families. USEARCH 6.0.203 [55] followed to compare the translated *de novo* set with the ORFs of CPRD to classify repeats at the superfamily level. ORFs were identified using the *findorfs* utility in USEARCH with default recovery parameters. Finally, since REPET analyzes the structural motifs of each element through TEclassifier, the assignments provided by REPET were used to classify the repeats by Order. The high quality, novel repeats discovered were stored as a multiple sequence FASTA file, and referred to as the Pine Interspersed Element Resource (PIER).

To characterize sequences without a qualifying match against CPRD, clustering techniques were applied to form groups of unique elements. These were subsequently sorted into repeat element families through all versus all alignment. These unannotated sequences were clustered using USEARCH's *cluster_fast* utility set to 80% similarity. A consensus sequence was generated for each cluster through multiple sequence alignment in MUSCLE [56] and PILER. The coverage of a family was determined by summing the number of base pairs of all full-length sequences belonging to all clusters within the family. The cluster with the most full-length sequences was selected as a representative for further analysis. Multiple sequence alignments of clusters representing high coverage families were visualized in Jalview [57], and the consensus sequence of each cluster was extracted. The consensus of each cluster was analyzed using BLASTN to target the LTR regions, and annotated using LTRdigest [58] with Pfam profiles [59]. In the challenging case of one nested, high-copy element, *PtOzark*, PILER proved superior at generating an informative consensus. A database of *Populus trichocarpa* tRNAs [60] was used to find matches to known repetitive element-associated proteins. Consensus repeats of ten novel, high-copy clusters were run in CENSOR with the normal sensitivity setting against four plant genomes available through Phytozome [41]: *Arabidopsis thaliana* v1.6.7, *Vitis vinifera* v1.4.5, *Cucumis sativus* v1.2.2, and *Populus trichocarpa* v2.1.0. Two conifer BAC resources were also compared: *Taxus mairei* [33] and *Picea glauca* [61] (Genbank Accession Nos: 262263013, 221149199; 327410373- 327412295). All references were evaluated to determine whether similar elements exist in other plant species.

### Gene Identification

A combination of similarity and *ab initio* gene prediction approaches were implemented to characterize the coding region of the BAC and fosmid sequence sets. In order to train *ab initio* gene prediction algorithms, a set of high confidence transcripts were extracted from the Newbler version 2.3 (454 Life Sciences, Branford, CT) assemblies of four Pinaceae species (*Pinus taeda*, *Pinus lambertiana*, *Pinus palustris,* and *Picea abies*) [14]. Full-length loblolly pine mRNA sequences available in Genbank were also included. Direct evidence for exon prediction was collected from ESTs and cDNAs of the following *Pinus* species closely related to loblolly pine, according to chloroplast phylogenetic analysis [62]: *P. ponderosa*, *P. attenuata*, *P. contorta*, *P. banksiana*, *P. thunbergii*, *P. resinosa*, *P. pinaster*, *P. elliottii*, *P. sylvestris*, *P. radiata*, *P. patula*, *P. torreyana*, and *P. palustris*. Alternative EST and cDNA evidence was provided by species further diverged, including: *Pinus lambertiana*, *Picea abies*, *Picea glauca*, *Picea sitchensis*, *Pseudotsuga menziesii* and *Picea mariana*. These transcripts were combined, and redundant sequences removed using USEARCH set to 95% identity. The resulting consensus sequences were used to train gene identification parameters for Augustus [63] inside of MAKER2 [64]. Identification of genes from the eukaryotic orthologous proteins (KOGS) was performed via CEGMA [65]. Repeat masking was performed in MAKER2 via Repeatmasker [66] and used the PIER library.

## Results

### Sequence sets

The cumulative base pair lengths of the BAC and fosmid libraries are 11,858,447 and 265,480,119, respectively. Combined, these resources represent approximately 1.26% of the estimated 22Gbp *Pinus* genome (Table 1). The molecular technologies used in library construction and sequencing gave rise to different read lengths, and, subsequently, assemblies, between the two sets. There was a three fold difference in their respective N50 lengths (127,167 bp for the BACs and 16,205 bp for the fosmids). Average GC content across the BACs was 37.98%, and 38.09% in the fosmids. The GC content was higher than in other conifers (*Taxus mairei* with 37.05% and *Picea glauca* with 37.11%) and dicots (*Juglans regia* L. with 37.7% [67], *Cucumis sativus* with 32.25%, *Vitis vinifera* with 34.55%, and *Populus trichocarpa* with 33.75%), but lower than in sequenced monocots, such as *Oryza sativa* (43%) [68].

**Table 1:** BAC and Fosmid Sequence Set Summary.

	BACs	Fosmids
**Total assembled sequences**	103	90,954
**Total length (bp)**	11,858,447	265,480,119
**Average sequence length (bp)**	115,130	2,918
**Median sequence length (bp)**	118,782	475
**N50 sequence length (bp)**	127,167	16,205
**Shortest sequence length (bp)**	1,392	201
**Longest sequence length (bp)**	235,088	75,791
**GC Content**	37.98%	38.09%
**Genbank Accessions**	GU477256-GU477266, AC241263-AC241362	APFE01000000

Unresolved nucleotides,‘N’, were not counted.

### Tandem repeats

A total of 63,622 tandem repeats were identified in the BACs and fosmids (Table S3). Combined, they covered 7.2 Mbp of the sequence sets (2.60%) (Table 2). Comparative analysis of other plant genomes revealed species with higher tandem content estimates including: *Vitis vinifera* (4.44%), *Populus trichocarpa* (4.24%), and the *Picea glauca* BACs (3.54%). Others were shown to have a lower tandem estimate, including: *Cucumis sativus* (1.56%) and the fosmid sequences of *Taxus mairei* (1.54%). The average length of an annotated tandem repeat array in loblolly pine was 116.72 bp in the BACs and 113.34 bp in the fosmids. Copies of repeat units ranged from 1.8 to 1,780 copies, with an average length of 6 bp and 4.4 bp in the BACs and fosmids, respectively. Minisatellites made up the largest physical portion of the genome (51,997 loci, 1.56%), followed by satellites (6,482 loci, 0.96%), and microsatellites (5,143 loci, 0.09%) (Table 2, Table 3). Copy number, however, was skewed towards microsatellites, with an average of 25 per array, 10 times that of either the minisatellites or satellites. Within the microsatellites, dinucleotides dominated, making up 74.5% of all the microsatellites. The (AT/TA)n motif alone had 1,869 arrays (38,976 copies, 47% of microsatellites), and constituted the largest physical area of the genome for microsatellite motifs, at 0.03%. AT-rich trinucleotides (ATT/AAT/ATA/TTA/TAT/TAA)n also made up a significant portion of the total microsatellites, with 347 arrays and 5,328 copies (8.72%). Interestingly, the single array with the highest copy number was a 25 bp period (TGCTTTGCTGCTTAGTCTCTCATAG) with 654 copies (∼16 Kbp) in the fosmid contig Pita_fosmid_APFE01000000_90533. This novel array, *Pita_MSAT16,* had no significant similarities when compared against the nucleotide (nt) database of NCBI and Repbase. After filtering for redundancy, tandem repeats annotated 3.31% of the BACs (3.04% of the 11 Sanger sequenced BACs) and 2.59% of the fosmids. However, after removing those that overlapped with interspersed content, these estimates were reduced to 0.93%, 0.53% and 0.54% of the BACs, fosmids and combined sets, respectively (Table 4).

**Table 2:** Summary of tandem repeats from BAC and fosmid sequences.

	Total loci	Copy number	Variants	Total length in bp (% of sequence sets)
**Microsatellites (2–8 bp)**				
** Dinucleotide**	2,967	64,740	10	126,254 (0.046%)
** Trinucleotide**	645	9,657.7	39	28,440 (0.010%)
** Tetranucleotide**	282	3,899.3	46	15,316 (0.006%)
** Pentanucleotide**	172	3,167.2	75	15,560 (0.006%)
** Hexanucleotide**	402	3,427.8	172	20,303 (0.07%)
** Heptanucleotide**	499	4,174	153	28,765 (0.010%)
** Octanucleotide**	176	920.1	135	7,184 (0.003%)
** Total**	**5,143**	**89,986.1**	**630**	**241,822 (0.09%)**
**Minisatellites (9–100 bp)**				
** 9–30 bp**	31,363	84,572.9	26,428	1,631,083 (0.588%)
** 31–50 bp**	11,800	30,641.9	10,989	1,164,786 (0.420%)
** 51–70 bp**	5,316	13,775.3	5,192	805,210 (0.290%)
** 71–100 bp**	3,518	8,642.9	3,473	722,282 (0.260%)
** Total**	**51,997**	**137,633**	**46,082**	**4,323,361 (1.559%)**
**Satellites (>100 bp)**				
** 101–200 bp**	5,183	12,589.3	5,110	1,710,062 (0.617%)
** 201–300 bp**	857	2,141.2	854	524,329 (0.189%)
** 301–400 bp**	280	691.6	280	236,623 (0.085%)
** >400 bp**	162	405.9	162	179,726 (0.065%)
** Total**	6,482	15,828	6,406	2,650,740 (0.956%)
** Grand Total**	**63,622**	**243,447.1**	**53,118**	**7,215,923 (2.602%)**

**Table 3:** Most frequent periods for three categories of tandem repeats in conifer genomic sequence.

	Pinus taeda (BAC + Fosmid)			Picea glauca (BAC)			Taxus mairei (Fosmid)		
	Micro	Mini	Sat	Micro	Mini	Sat	Micro	Mini	Sat
**Most frequent period**	2	21	123	2	27	122	2	24	230
**Cumulative length**	126,254	216,194	154,835	876	843	389	1,411	587	1,598
**Num. of loci**	64,740	10,508	1,258	11	10	1	32	19	2
**Most frequent period (%)**	0.05%	0.08%	0.06%	0.33%	0.32%	0.15%	0.09%	0.04%	0.10%
**Total cumulative length (bp)**	241,822	4,323,361	2,650,740	925	6,603	1,864	3,024	15,875	5,871
**Total (%)**	**0.09%**	**1.56%**	**0.96%**	**0.35%**	**2.49%**	**0.70%**	**0.19%**	**0.98%**	**0.36%**

Micro – Microsatellites (2–8 bp).

Mini – Minisatellites (9–100 bp).

Sat – Satellites (>100 bp).

**Table 4:** Summary of full-length repetitive content.

	BAC		Fosmid		Combined	
	Similarity	De novo	Similarity	De novo	Similarity	De novo
**Class I**	2.9376%	33.682%	1.3394%	21.295%	1.3879%	21.8222%
** LTR retrotransposon**	2.9376%	25.1368%	1.3388%	14.8972%	1.3873%	15.3333%
** Gypsy**	2.0772%	2.4524%	0.918%	1.1181%	0.9477%	1.175%
** IFG7**	0.5793%	1.1027%	0.2189%	0.3109%	0.2184%	0.3447%
** Gymny**	0.1541%	0.3491%	0.0897%	0.0942%	0.0924%	0.1051%
** Corky**	0.9716%	0.0543%	0.3894%	0.1743%	0.4143%	0.1692%
** PGGYPSYX1**	0.3722%	0%	0.22%	0%	0.2226%	0%
** Other**	0%	0.9462%	0%	0.5387%	0%	0.556%
** Copia**	0.8604%	1.6113%	0.4208%	0.5343%	0.4396%	0.5803%
** TPE1**	0.8422%	0.8066%	0.4072%	0.3699%	0.4258%	0.3885%
** TY1_PE**	0%	0.5255%	0%	0%	0%	0.0225%
** Copia4-PTR**	0%	0%	0.0073%	0.0096%	0.007%	0.0092%
** Copia_ES**	0%	0%	0.0002%	0%	0.0002%	0%
** RT_GB**	0.002%	0%	0.0003%	0%	0.0003%	0%
** RT_PT**	0.0162%	0%	0.0058%	0%	0.0063%	0%
** Other**	0%	0.2792%	0%	0.1549%	0%	0.1602%
** DIRS**	0%	0.2988%	0%	0.2213%	0%	0.2246%
** PLE**	0%	0.1665%	0%	0.1266%	0%	0.1283%
** LINE**	0%	1.4017%	0.0003%	0.6752%	0.0003%	0.7062%
** PILN1_PT**	0%	0%	0.0003%	0%	0.0003%	0%
** SINE**	0%	0.0162%	0%	0.0025%	0%	0.003%
**Class II**	0%	1.2173%	0%	0.5023%	0%	0.5328%
** TIR**	0%	1.0497%	0%	0.3276%	0%	0.3584%
** Helitron**	0%	0.0221%	0%	0.038%	0%	0.0373%
**Annotated**	100%	2.8383%	100%	0.9588%	1%	1.0391%
**Total interspersed content**	2.9376%	38.8427%	1.3394%	25.4083%	1.3879%	25.9827%
**Tandem repeats** *	0.9292%		0.5224%		0.5398%	
**Total repetitive content**	**3.8668%**	**39.7719 %**	**1.8618%**	**25.9307%**	**1.9277%**	**26.5225%**

* TRF estimates, non-overlapping with interspersed content.

**Figure 2:**
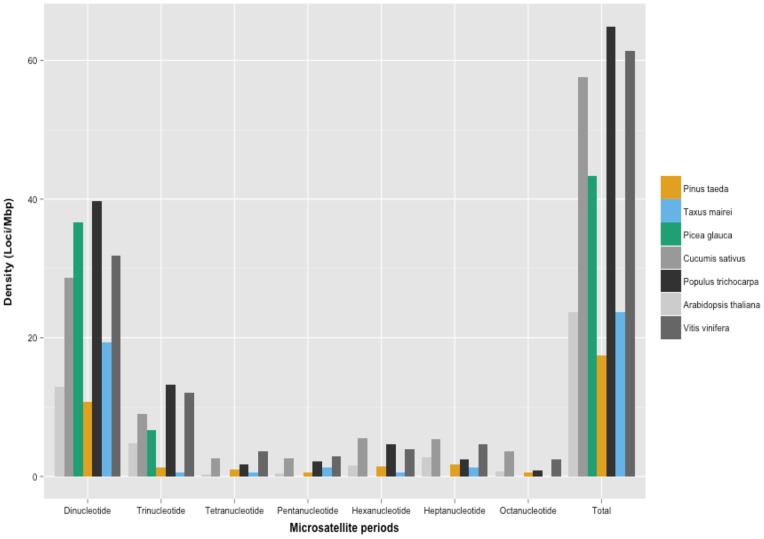
Microsatellite density across multiple species. Cross-species comparison of microsatellites ranging from dinucleotide to octanucleotide, as calculated by TRF (microsatellite/Mbp). Analysis included two gymnosperm BAC sets (*Picea glauca, Taxus mairei*) and four angiosperms genomes (*Cucumis sativus, Arabidopsis thaliana, Vitis vinifera, Populus trichocarpa*).

Among species compared, microsatellite densities, from highest to lowest, were as follows: *Populus trichocarpa, Vitis vinifera, Cucumis sativus, Picea glauca, Taxus mairei, Arabidopsis thaliana,* and *Pinus taeda* (Figure 2, Table S2). In comparisons with other species, the ratio of minisatellites to satellites was large, as expected (*Pinus taeda*: 8.1, *Arabidopsis thaliana:* 10.5, *Cucumis sativus:* 27, *Vitis vinifera: 6.2, Populus trichocarpa:* 6.2, *Picea glauca:* 17.33, *Taxus mairei:* 13.29). Minisatellites of 21 bp were most common, with the exception of *Picea glauca*, in which 27 bp lengths were more common. Average minisatellite lengths ranged from 66.69 bp (*Arabidopsis thaliana*) to 94.33 bp (*Picea glauca*), with *Pinus taeda* at 83.7 bp. Average satellite length varied significantly between angiosperms, from 350.55 bp (*Cucumis sativus*) to 931.28 bp (*Arabidopsis thaliana*). Among the gymnosperms, satellite lengths were similar, and averaged 419.4 bp in *Taxus mairei*, 424.5 bp in *Pinus taeda,* and 466 bp in *Picea glauca*.

Potential centromeric, telomeric, and heterochromatic repeats were identified using Censor [42], though most were spurious, with an average coverage of 28% and a maximum of 75% identity. Of these spurious matches, 247 fragments were from *Zea mays*, four from *Zingeria biebersteiniana,* one from *Sorghum bicolor*, and one from *Secale cereale*. The previously characterized plant telomeric repeat (TTTAGGG)n [69] was detected a total of 237 times across 23 different arrays.

### Interspersed Repeat Identification (Homology)

**Figure 3:**
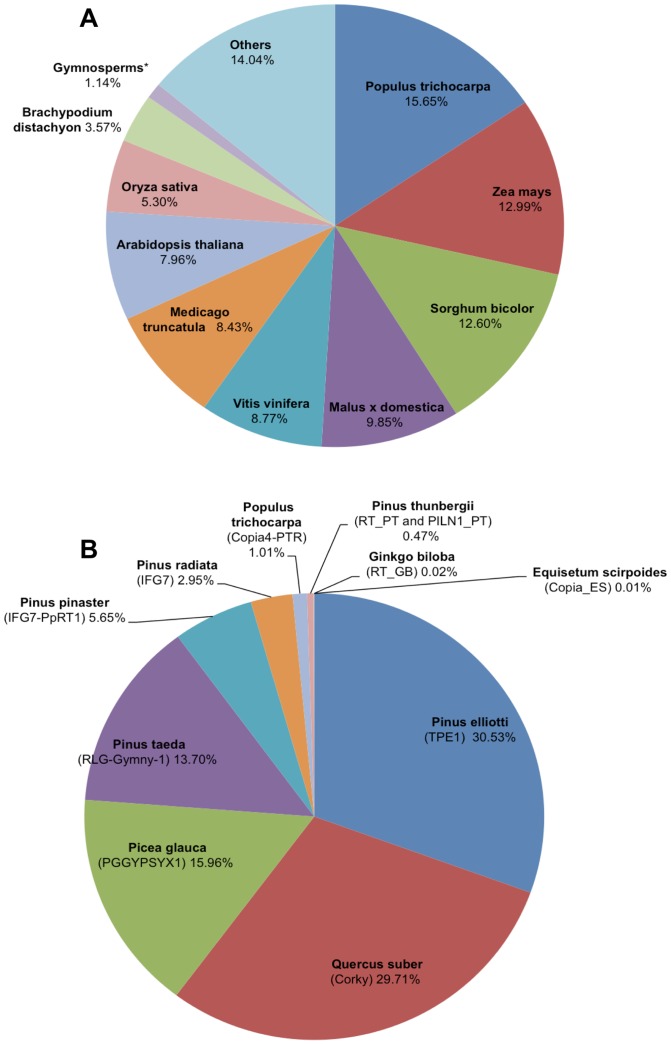
Distribution of homology-based repeat annotations by species. Interspersed repeats were analyzed via a redundant similarity search (CENSOR against CPRD). Percentage in each sector represents base pair coverage over the redundant annotations. **(A)** Displays species coverage for full-length and partial elements. Species with contributions less than 3%, were categorized as ‘Other’. **(B)** Displays species coverage for full-length elements only.

Homology searches against Repbase, using Censor, yielded a total of 14,470 and 175,004 hits to repeat fragments in the BACs and fosmids, respectively. A large portion of elements annotated by unfiltered Censor runs were previously characterized in *Populus trichocarpa* (15.7% of alignments), followed by *Zea mays* (13.0%), *Sorghum bicolor* (12.6%) and *Malus domestica* (9.9%) (Figure 3A). After filtering for full-length copies, 94 were identified in the BACs and 989 in the fosmids. Of these, 69.25% were previously characterized in conifers, the majority annotated as the Copia element, *TPE1*, originally characterized in *Pinus elliottii* (30.53%) (Figure 3B). These filtered alignments represent 2.94% of the BAC sequence and 1.34% of the fosmid sequence (Table 4). When accounting for both partial and full-length hits, 57 unique repeat families spanning 14 repeat orders aligned to the fosmid set. The BACs aligned to 28 distinct families spanning 11 orders. All of the families identified in the BACs were also present in the fosmids. Both sets contained elements from each of the two transposable element classes, Class I (retrotransposon) at 20.41% and Class II (DNA transposon) at 4.03%. The most prevalent superfamilies (Class: Order: Superfamily), based on the number of unique copies (>500 in the fosmids and BACs) from Class I, included LTRs (Gypsy and Copia), Non-LTR L1 (LINEs), other LTRs, and Caulimoviridae (an integrated virus). Among Class II, terminal inverted repeats (TIR), EnSpm, and Helitrons were present. The TIRs annotated included MuDR (0.80% of the sequence sets), hAT (0.74%), EnSpm (0.72%), Mariner/Tc1 (0.32%), and Harbinger (0.14%) (Table S5).

**Figure 4:**
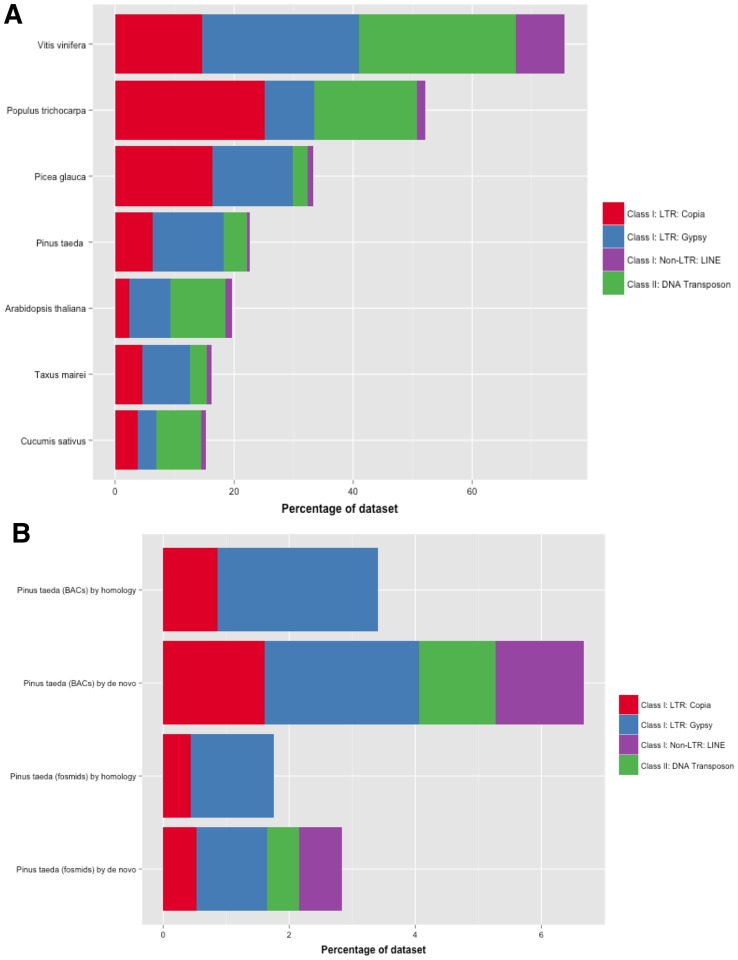
Distribution of transposable elements from similarity search. A combination of the non-redundant CENSOR results from the BAC sequences (103) and fosmid sequences (90,954) were used to ascertain the major contributing classes of TEs. **(A)** Compares partial and full-length TE content by homology against other species. **(B)** Examines the full-length TE content in loblolly pine annotated in homology based and *de novo* searches.

The estimate of partial and full-length repetitive content by homology of *Pinus taeda* is 27.50% (Table 5). The primary contributions include 6.27% Copia elements, 11.97% Gypsy elements, 3.95% DNA transposons and 0.49% LINEs (Table S5). This estimate is slightly lower than *Picea glauca* (33.24%), *Populus trichocarpa* (33.91%), and *Vitis vinifera* (42.03%) (Figure 4A). Among the full-length hits, Gypsy represented 2.54% and 1.32% of the sequence in the BACs and the fosmids, respectively. Copia represented 0.86% and 0.43% (Figure 4B). Alignments with high overall similarity and coverage (88.75% and 93.87%, respectively) to ribosomal RNA were detected in the BACs and represented 0.22% of the sequence set. Two 414 bp PILN1 LINE elements previously characterized in *Pinus thunbergii* were identified in the fosmids.

**Table 5:** Filtered (full-length) vs. Unfiltered (partial and full-length) repetitive content estimates.

	P + F (Homology)	Filtered (Homology)	P + F (de novo)	Filtered (de novo)
**Class I**	20.41%	1.39%	73.39%	21.82%
**Class II**	4.03%	0%	1.52%	0.53%
**Other**	3.06%	2.6%	10.91%	4.16%
**Total**	**27.50%**	**3.99%**	**85.82%**	**26.51%**

Seven conifer-specific TEs, including: IFG7-PpRT1 from *Pinus pinaster* [44], PGGYPSYX1 from *Picea glauca* [19], TPE1 from *Pinus elliottii* [43], PtIFG7 from *Pinus taeda* [7], IFG7_I from *Pinus radiata* [70], PGCOPIAX1 from *Picea glauca* [19], and RLG_Gymny_1 from *Pinus taeda* [3], along with one *Quercus suber* TE, Corky [45], were prevalent. TY1_PE from *Pinus elliottii* and RT_PT from *Pinus thunderbergii* were found in the BACs and fosmids at similar frequencies. Though not the highest in copy number, Corky annotated the largest physical portion of the BACs (almost 1%) and a significant portion of the sequence set (0.41%) (Table 4). The fosmid scaffolds produced significant alignments with IFG7 from *Pinus radiata.* This LTR aligned 23 times with an average similarity of 88.8% and coverage of 91.5% to the internal portion, and nine times with an average similarity of 91.4% and coverage of 99.1% to its LTRs. IFG7's complete presence was estimated at 0.58% in the BACs and 0.22% in the fosmids (Table 4). Copia4-PTR_I from *Populus trichocarpa* aligned four times to the internal portion (average similarity of 90% and coverage of 89.7%) with no full-length hits corresponding to the LTRs (Table 4). The longest alignments in both the BACs and the fosmids were to full-length, presumably autonomous, RLG_Gymny-1 elements [3]. In the BACs, three of these elements were discovered, with an average similarity of 80% and 100% coverage against the consensus. In the fosmids, there were 11 alignments, with an average similarity of 82% and 100% coverage. The Copia element, TPE1, was the highest in copy number in both sets, with 26 alignments in the BACs (average similarity 94.4%, average coverage 98.6%) and 287 in the fosmids (average similarity 94.4%, average coverage 98.6%). In addition, TPE1 was the highest in overall coverage in the fosmids, with 0.4% coverage (Table 4). PGGYPSYX1 from *Picea glauca* was also discovered (Figure 3B, Table 4). Homology searches for full-length elements in the selected angiosperms (*Arabidopsis thaliana, Cucumis sativus, Populus trichocarpa, Vitis vinifera*) yielded less informative results.

### Interspersed Repeat Identification (de novo)

The consensus sequences generated from the REPET pipeline were used as seeds to validate the repeats against the original BAC and fosmid sequences. Initial self-alignment in REPET resulted in 3,433 unfiltered hits in the BAC sequences and 1,654,975 unfiltered hits in the fosmid sequences. Clustering with Grouper, Recon, and Piler resulted in 166, 139, and 27 clusters, respectively, in the BACs and 11,405, 3,814, and 10,33 clusters, respectively, in the fosmids. Among the BACs, 1,256 high-scoring segment pairs (HSPs), representing 325 seeds, and covering 21.64% of the BAC sequence, were annotated as LTRs. In the fosmid set, 60,467 HSPs, representing 5,061 seeds covering 13.95% of the fosmid sequence, were annotated as LTRs. Combined, the LTR content spans 61,723 HSPs constructed from 5,386 seeds, and covers 14.28% of the sequence set. 1,518 HSPs covering 28.74% of the BAC sequence were built from alignments of 488 Class I seeds against BAC sequences. 70,860 HSPs covering 19.54% of fosmid sequence were built alignments of 8,097 Class I seeds against fosmid sequences. The total Class I content for both sets is represented by 72,378 HSPs built from 8,585 seeds, and covers 19.94% of the sequence set. Seeds annotated as Class I retrotransposons included LTR content. Comparatively few seeds were annotated as DNA transposons: only 0.66% of the BAC sequence corresponding to 13 seeds and 0.23% of the fosmid sequence corresponding to 155 seeds, for a combined total of 0.25% (168 seeds). Uncategorized sequence accounted for 81 seeds and 842 HSPs totaling 3.26% of BAC sequence and 3,034 seeds and 75,060 HSPs totaling 4.68% of fosmid sequence. In all cases, BAC-derived HSPs were longer than fosmid-derived HSPs, and the ratio of HSPs to seeds for each category was larger in the BACs than in the fosmids. In the BACs, 2,444 HSPs representing 591 seeds covered a total of 32.94%. 5,061 HSPs representing 11,631 seeds covered 24.93% of the fosmid sequence. With the BAC and fosmid sequence sets combined, 155,686 HSPs representing 12,222 repeat seeds covered 25.27% of the sequence (Table S6).

The final set of non-redundant repeat seeds (12,222), with 11,631 from the fosmid set and 591 from the BAC set, returned alignments against 15,747 full-length sequences across both datasets (Table S6). These sequences had an average length of 4,414 bp and represented 25.98% of the sequence. 489 of these could be classified as one of the six characterized Gypsy or Copia families IFG7, Gymny, Corky, TPE1, Copia4-PTR_I, or TY1_PE, and represent 1.04% of the sequence set. Of the remaining 15,258 sequences, none aligned with confidence to families in CPRD. 11,119 of these sequences, however, could be classified manually at various resolutions. These repeats had an average length of 5,577 bp and represented 22.36% of the sequence set. At the *Class* level, 10,431 sequences, with an average length 5,803 bp, were classified as retrotransposons, while 688, with average length 2,148 bp, were classified as DNA transposons, covering 21.82% and 0.53% of the sequence set, respectively (Table S6). At the *Order* level, LTRs composed the bulk of the repetitive content, with 6,666 sequences representing 15.3% of the sequence set. DIRS, Penelope, and LINE elements represented a small portion of *Class I* elements, with coverage of the combined sequence. Within *Superfamily*, 617 sequences with an average length of 5,282 bp were annotated as Gypsy, and 317 sequences with an average length 5,078 bp were annotated as Copia. They represented 1.18% and 0.58% of the sequence set, respectively. Unclassified sequences composed 3.62% of the sequence sets (13.95% of the repeats), with an average length of 2,172 bp. These sequences did not align significantly against known sequences, and TEclassifier was unable to assign annotations to them.

**Figure 5:**
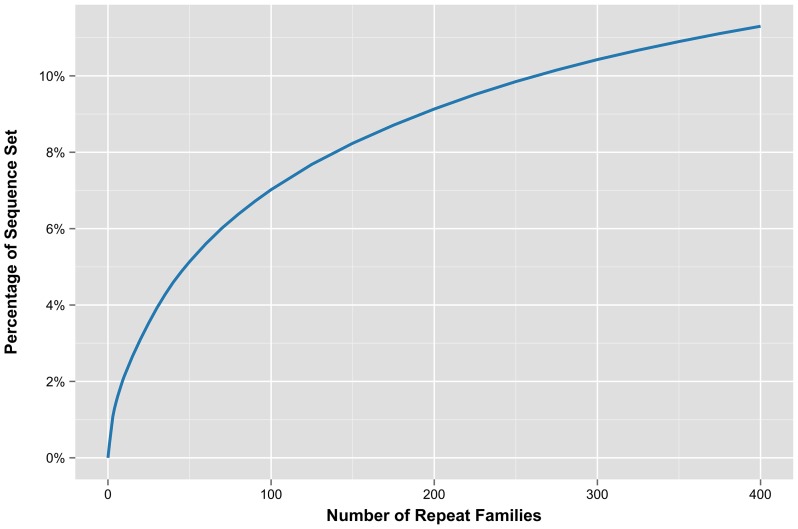
Genomic sequence represented by the highest coverage elements. Base pair coverage attributed to copies of the high coverage LTR TEs.

Clustering of all unannotated sequences yielded 9,415 clusters (subfamilies), of which 7,015 were singletons, 1,357 contained two sequences, 471 contained three sequences, 195 contained four sequences, and 125 contained five sequences. The top 1% of clusters each contained at least nine full-length sequences. All versus all alignments resulted in 6,270 families. 5,155 elements were considered single-copy families, and the remaining 3,125 clusters were grouped into 1,115 families. In total, 10,057 elements were grouped into families, while 5,155 elements remained single-copy. As a result of the all versus all alignment, 559 full-length elements were grouped into the ten highest-coverage novel families, representing about 2% of the sequence (Table 6). Elements grouped into the top 100 highest coverage families (including known elements) account for about 19 Mbp (Table S7), or 7% of the sequence set, while the top 400 highest families account for over 11% of the sequence set (Figure 5). Sequences annotated as members of known repeat families account for most of the largest families when compared side by side with the *de novo* families. 159 elements annotated as TPE1 comprised 0.39% of the sequence set, 162 elements aligned to IFG7 represented 0.34% of the sequence set, 78 elements aligned to Corky accounted for 0.17% of the sequence set, and 24 elements aligned to Gymny comprised 0.11% of the sequence set (Table 6).

**Table 6:** High coverage LTR families identified with the *de novo* methodology.

Repeat family	Full-Length Copies	Length (bp)	Percent of Sequence Set
**TPE1**	159	1,077,598	0.39%
**PtPiedmont (93122)**	133	969,109	0.35%
**IFG7**	162	956,018	0.34%
**PtOuachita (B4244)**	47	576,871	0.21%
**Corky**	78	469,286	0.17%
**PtCumberland (B4704)**	67	431,492	0.16%
**PtBastrop (82005)**	38	378,631	0.14%
**PtOzark (100900)**	32	378,020	0.14%
**PtAppalachian (212735)**	67	367,653	0.13%
**PtPineywoods (B6735)**	68	322,632	0.12%
**PtAngelina (217426)**	24	309,248	0.11%
**Gymny**	24	291,479	0.11%
**PtConagree (B3341)**	50	285,850	0.10%
**PtTalladega (215311)**	33	274,826	0.10%
**Total**	**982**	**7,088,713**	**2.56%**

The top ten *de novo* LTRs and top four previously characterized families (as annotated through homology searches) account for over 7 Mbp of sequence, or 2.56% (Table 6). None of the novel *de novo* families annotated in this study have significant alignments, as defined by the 80-80-80 standard, against CPRD, or when compared against the other six plant genomic datasets. These ten represent six Gypsy elements, three Copia elements, and one unknown LTR. The largest family, *PtPiedmont*, is an LTR retroelement that contains 133 sequences from six different clusters across almost 1 Mbp of sequence (0.35%). The average sequence length of each element in the representative cluster of *PtPiedmont* is 7,340 bp. This element is characterized by LTRs that are about 1,000 bp long, and has a primer binding site (PBS) directly adjacent to the 3′ LTR. The absence of alignments in the internal region limits the possibility of assigning this element to a superfamily.

**Figure 6:**
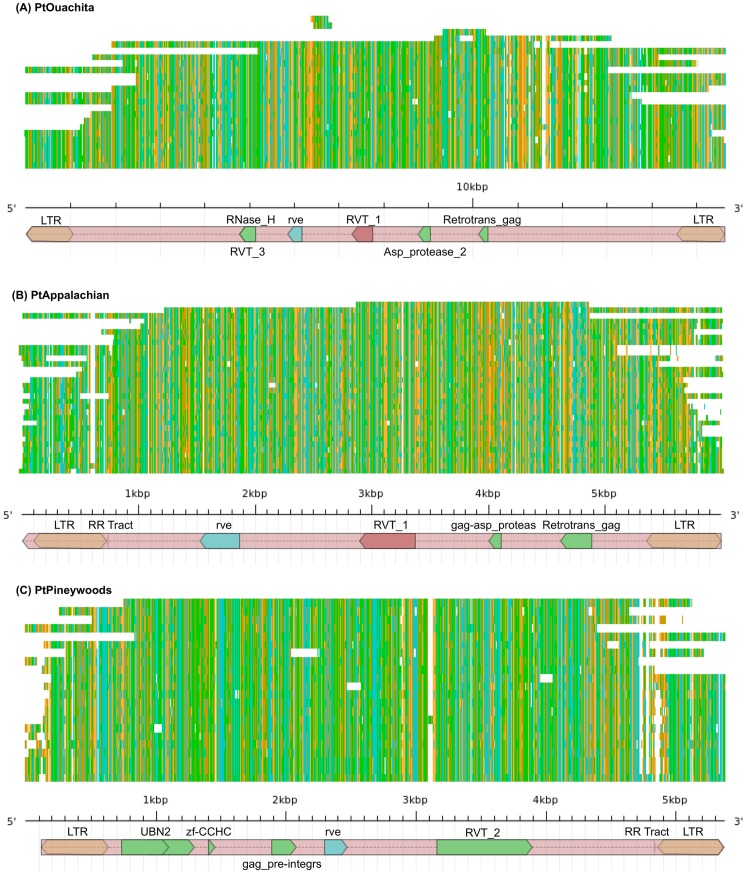
Annotated high copy LTR repeat families. Multiple alignments of the top ten high coverage and novel elements were performed using MUSCLE and visualized in Jalview. The final consensus sequence was exported with substitutions resolved, annotated (LTRdigest), and visualized (AnnotationSketch). **(A)** Multiple sequence alignment of the 24 sequences in the representative cluster of the *PtOuachita* family. **(B)** Multiple sequence alignment of the 67 sequences in the representative cluster of the *PtAppalachian* family. **(C)** Multiple sequence alignment of the 68 sequences in the representative cluster of the *PtPineywoods* family.

The six novel Gypsies include: *PtOuachita, PtBastrop, PtOzark, PtAppalachian, PtAngelina,* and *PtTalladega.* The second largest *de novo* family, *PtOuachita*, contains 47 sequences from 2 clusters across 577 Kbp, or 0.21%, of the sequence set. The average sequence length of *PtOuachita*'s representative cluster is 13,058 bp. This element is characterized by LTRs that are about 1,000 bp long and alignments to RNase_H, RVT_3, rve, RVT_1, Asp_protease_2, and Retrotrans_gag protein families (Figure 6A). *PtOuachita* aligns spuriously to LTR retroelements found in *Brachypodium distachyon*, *Sorghum bicolor*, *Physcomitrella patens*, *Vicia pannonica*, and *Arabidopsis thaliana*. Translated searches yield a 1,750 bp alignment to Gypsy-2_SMo-I at 40.3% similarity. *PtBastrop*, with 38 sequences covering 379 Kbp (0.14%) of the sequence set, is 15,520 bp long, and is characterized by a 15 bp primer binding site, LTRs that are about 1,100 bp long, and alignments to Retrotrans_gag, Asp_protease_2, RVT_1, rve, RNase_H, and RVT_3 protein families. *PtBastrop* aligns trivially to LTR retroelements found in *Populus trichocarpa*, *Vicia pannonica*, and *Medicago truncatula*. Translated searches yield a 2,750 bp alignment to Gymny at 38.7% similarity. *PtOzark* contains 32 elements and covers 378 Kbp, or 0.14% of the sequence set, and is 29,074 bp long. The majority of the sequence aligns with itself. Two 13.4 Kbp regions, which both encompass almost half of the total sequence, align to each other at over 90% identity, and contain similar LTRs. A portion of *PtOzark*'s internal sequence could be identified as a second, nested retroelement that contains LTRs of approximately 350 bp that align at 81% identity. This putative nested retroelement also contains alignments to rve, RVT_1, RVT_3, gag-asp_proteas, and Retrotrans_gag protein families. The full element is characterized by LTRs that are about 50 bp long, a 5′ 18 bp PPT, and a gag-asp_proteas protein family alignment in a region outside of the putative internal element (Figure S1). *PtOzark* aligns trivially to many LTR elements in over ten species in CPRD. Translated searches yield a 1,450 bp alignment to Gypsy-2_BD_I at 39% similarity. *PtAppalachian*, with 67 full-length copies covering 368 Kbp (0.13%) of the sequence set is 5,995 bp long and characterized by LTRs that are 620 bp long. It has been annotated with a 5′ 13 bp PPT, and aligns to rve, RVT_1, gag-asp_protease, and Retrotrans_gag protein families. *PtAppalachian*'s consensus sequence extends beyond the 5′ LTR by 96 bp. This element aligns to a 2.2 Kbp region to the Gypsy, PGGYPSYX1 at 90.3% similarity (Figure 6B). *PtAngelina*, with 24 full-length copies covering 309 Kbp (0.11%) of the sequence set, is 15 Kbp long, and is characterized by LTRs that are about 1,020 bp long and alignments to RVT_3, RNase_H, rve, RVT_1, Asp_protease_2, and Retrotrans_gag protein families. Translated searches yield a 2,070 bp alignment to Gypsy-2_SMo-I at 39.4% similarity. *PtTalladega*, with 33 full-length copies, covering 275 Kbp (0.10%) of the sequence set, is 15,387 bp long. It is characterized by LTRs that are approximately 1,000 bp in length and alignments to RVT_3, RNase_H, rve, and RVT_1 protein families. *PtTalladega* aligns trivially to LTR retroelements found in *Oryza sativa*, *Medicago truncatula*, *Vitis vinifera*, and *Zea mays*. Translated searches yielded a 2.5 Kbp alignment to Gymny at 40.4% similarity.

Three novel elements were characterized as Copia LTRs. The third largest novel family, *PtCumberland*, with 67 sequences covering 431 Kbp (0.16%) of the sequence set, has a length of 9,092 bp. This element is characterized by LTRs that are about 1,500 bp long, an 11 bp polypyrimidine tract (PPT) adjacent to the 5′ LTR, and alignments to RVT_2, rve, gag_pre-integrs, zf-CCHC, and UBN2 protein domains. *PtCumberland* aligns to GmCOPIA10 for 3 Kbp at 67.4%. *PtPineywoods*, with 68 full-length copies covering 323 Kbp (0.12%) of the sequence set, is 5,373 bp long, and is characterized by LTRs that are 510 bp long, a 3′ PPT, and alignments to UBN2, zf-CCHC, gag_pre-integrs, rve, and RVT_2. Translated searches yield a 1,600 bp alignment to Copia-4_PD-I at 59.2% similarity (Figure 6C). *PtConagree*, with 50 full-length copies covering 286 Kbp (0.10%) of the sequence set, is 15,552 bp long, and is characterized by 1,060 bp LTRs. It is annotated with a 3′ PBS, and alignments to RVT_3, RNase_H, rve, RVT_1, Asp_protease_2, and Retrootrans_gag protein families. *PtConagree* aligns across a 4 Kbp to Copia-31-I_VV at 70.3% similarity.

### Gene Identification

Of the 458 Conserved Eukaryotic Genes considered in CEMGA, only 23 full-length (70% similarity) genes were present in the BAC and fosmid sequence sets. Transcription factors, heat shock, and ribosomal proteins were among the categories represented. The majority of these identifications were found in the fosmid sequences; just one of the 23 was identified in the BAC set. Analysis of the full set of plant orthologous proteins provided for an additional eight full-length proteins. Three of the eight were exostosin proteins thought to be involved in cell-wall synthesis. Other annotations included an aquaporin, ATP synthase, pyrophosphorylase, and two hypothetical proteins. These orthologous proteins were identified in a range of species including *Zea mays*, *Medicago truncatula*, *Glycine max*, *Oryza sativa*, *Populus trichocarpa*, and *Ricinus communis*. Augustus provided the *de novo* gene identifications, and a total of 21 well-supported and full-length identifications were confirmed. The combined 52 genes identified have lengths ranging from 3 Kbp to just over 8 Kbp, contributing to the annotation of 298 Kbp of sequence. The intron sizes were overall small, all less than 2 Kbp in length. The substantial annotation of genic content given the small percentage of the genome analyzed could be reflective of the pseudogenes known to be prevalent in conifers.

### Data Availability

The multi-FASTA CPRD Repbase library, as well as the library generated from this study, PIER, are available at TreeGenes [13] for download (http://dendrome.ucdavis.edu/resources/downloads.php). The annotated fosmid and BAC sequences can be viewed through GBrowse, also hosted at TreeGenes (http://dendrome.ucdavis.edu/treegenes/gbrowse/). The ten novel repeats characterized here have been submitted to Repbase. All fosmid sequences have been submitted to Genbank as WGS: APFE01000000.

## Discussion

We performed an extensive characterization of repetitive elements in loblolly pine with sequence constructs representing just over 1% of the estimated 22Gbp loblolly pine genome. Our estimate of the total repetitive content comes in two flavors: an estimation that considers all partial alignments to known or de novo repetitive elements (about 86%), and an estimation that considers only elements that satisfy the 80-80-80 rule (about 27%) (Table 5). Few studies have explored repetitive content by using full-length elements to quantify the relative frequencies of different families. As in previous studies, we noted that LTRs dominated the repeat landscape, contributing to over 60% of the overall repetitive content. As seen in most plant genomes, the Gypsy and Copia LTRs were most prevalent. Our *de novo* investigation was able to identify a multitude of LTRs that have not been characterized in conifers and showed very little similarity to elements characterized in other species. In addition, we annotated 52 full-length genes through orthologous and de novo techniques that cover approximately 298 Kbp. Here, we discuss these novel contributions to the expansive genome.

### Tandem repeats

Tandem repeats are traditionally divided into three classes: microsatellites, minisatellites, and satellites. They can arise from polymerase slippage, can serve as recombination hotspots, act as effectors of gene expression, and are linked to variation [69], [71]–[73]. While the total tandem content for loblolly pine was estimated at 2.6%, it should be noted that the BAC sequences alone had an estimated content of 3.3%, which is much closer to the estimate of 3.4% for the *Picea glauca* BACs. The large difference between the fosmids and BACs could be explained by the different scaffold lengths, sequence types, and assembly technologies used. A subset of the *Pinus* BACs were assembled from Sanger data, which is less likely to experience the repeat collapse common in short read assemblies [74].

Microsatellites (simple sequence repeats) are characterized by a repeat unit of 1–8 bp. They are numerous in genomes and are useful as genetic markers due to their polymorphisms [75]. Loblolly pine had a low microsatellite density when compared to other species within and outside the conifer division (Table 2), a finding consistent with previous analyses [76]. For example, *Vitis vinifera* and *Picea glauca* are 1.8 to 2.6 times richer in microsatellite content. Dinucleotide repeats lead in density for every species compared. AT/TA motifs ranged from 42.04% of the microsatellite arrays in *Vitis vinifera* to 55.03% in *Cucumis sativus*. In all but *Taxus mairei*, trinucleotides followed, with an average density of 18.3 (microsatellite/Mbp). The third densest microsatellites for *Pinus taeda, Arabidopsis thaliana, Vitis vinifera,* and *Taxus mairei* were heptanucleotides. Most significantly, *Pinus taeda* and *Arabidopsis thaliana* heptanucleotide arrays make up 16% and 17.2% of their respective microsatellite arrays. Microsatellite density is generally higher in intergenic regions and introns than in genic regions [77], which supports their role in gene regulation. However, the low microsatellite density in loblolly pine is likely not due to higher genic content, but to the prevalence of more complex repeats, such as interspersed retrotransposons.

Minisatellites (repeat unit between 9 and 100 bp) evolve quickly and are predominantly GC-rich. Both micro- and minisatellites exhibit length/copy number polymorphisms, and originate in similar ways. Certain period sizes, especially 20–25 bp (Table 2), are prevalent within *Pinus taeda*, suggesting they may be conserved. One hypothesis states that not only are these repeats highly conserved, but also species specific [76], [78]. Longer satellites and minisatellites have been observed to have unique hybridization patterns when compared between *Picea* and *Pinus* [76]. Our discovery of a ∼16 Kbp minisatellite, *Pita_MSAT16* (Table S4), with a period size of 23 bp, had no significant homology to previously annotated tandem repeats.

Satellites (>100 bp) are prevalent in centromeres, telomeres, and heterochromatin. The distribution of perfect satellites in the five angiosperms differed from previous studies such as [77] and [23]. The methodologies employed to determine these estimates also varies in these studies. Among the gymnosperms examined here, significant variation was noted. The most prevalent satellite from *Taxus mairei* had 230 bp periods, almost double the size of those in *Pinus taeda* (123 bp) and *Picea glauca* (121 bp) (Table 3). Regions that returned spurious homologies to known telomeric sequences may be due to telomere-like repeats that are highly amplified and form large intercalary and pericentric blocks [76]. Thus, they are present not only on the ends of chromosomes but as repeated components of similar structure elsewhere.

Our results found the (AT/TA)n motif to be most prevalent, while previous studies noted that the (AC)n and (AG)n motifs are most common in conifers [76], [79], [80]. The frequency of this motif was, however, less than those computed for *Vitis vinifera* (42.04%) and *Cucumis sativus* (55.03%). The (AT/TA)n motif was also one of the most common dinucleotides observed in papaya (*Carica papaya* L) [78]. AT-richness, defined as A%+T%>60% for any given sequence, prevailed in every class of tandem repeat across all species evaluated. In this study, minisatellites in *Pinus taeda* were 61.45% and 65.22% AT-rich in the BACs and fosmids, respectively. There is a slight trend for lower AT-richness in conifer micro- and minisatellites when compared to angiosperms as seen when *Pinus taeda* or *Picea glauca* (at 61.43% AT-richness) are compared against the 85.77% of *Cucumis sativus* (Table S3). AT-rich repeats prevail in dicots but not monocots [77], and apparently not in conifers. However, due to the limited availability of resources for other gymnosperms considered, it cannot be said with confidence that the tandem repeats identified show gymnosperm-specific patterns.

### Interspersed content

A typical investigation of repetitive content involves homology-based searches against a database of known repetitive elements. The primary repository for this purpose, RepBase, contains only 15 elements that have been characterized in gymnosperms. The custom database that we created, CPRD, includes another five elements from conifers described in the literature. These 19 and the full contribution of angiosperm elements in RepBase could only annotate 1.4% of the sequence set as full-length elements and represented just four of the top 14 high-coverage novel elements (Table 6). Even with partial element annotations, the total sequence attributed to tandem and interspersed repeats by homology is 28%. Based on the large genome size and knowledge of retrotransposon expansion, we expected a much higher estimate. A *de novo* approach was critical in describing the significant number of highly diverged retrotransposons. The REPET pipeline combines both sequence self-alignments and structural identifications to do this. The self-alignment portion uses three different local alignment/pattern detection packages (GROUPER, RECON, and PILER) as well as subsequent processes to reduce redundancy and identify a consensus sequence for each element [47]. The structural identification of LTR retrotransposons through LTRharvest is ideal for characterizing low or single-copy elements. The combination of similarity and *de novo* methods allowed us to identify 29% of the sequence as full-length elements and nearly 86% as full or partial. In short, we were able to apply both approaches to maximize the sensitivity and specificity needed to find and characterize diverged repeats.

The combined, full-length and partial element estimate of 86% falls just outside the range provided previously for loblolly pine (24% -80%) according to [7], who first examined ten of the 103 BAC sequences. It is comparable to the estimate in *Taxodium distichum* (90%), but much higher than that in *Picea glauca* (40%) [19], [20]. While this estimate is higher than most angiosperms, a few species including *Zea mays* (85%) and *Hordeum vulgare* (84%) [12], [81] are reported to have similar amounts of repetitive content. The estimate of full-length retrotransposons in the sequence sets surveyed was 22%, which represents about 87% of the full-length repetitive content. Many angiosperm species have comparable ratios for retroelements, including *Sorghum bicolor* (70–76%), *Zea mays* (88%), and *Glycine max* (72%) [82]. The full-length repetitive sequence captured with both similarity and *de novo* approaches was greater in the BAC sequences (39%) than in the fosmids (26%). In addition, both classes of TEs had over 1.5x the number of full-length identifications in the BACs when compared with the fosmids. The LTRs characterized had lengths up to 29 Kbp and could easily be missed in the smaller fosmid sequences. As mentioned previously, the Sanger sequenced BACs may also have a superior assembly in repetitive regions allowing for improved identification.

Two primary divisions exist to describe interspersed repetitive content, generally known as transposable elements. Class I retrotransposons require reverse-transcription. They can be divided into two types, based on the presence or absence of direct repeats at the ends of the element, known as long terminal repeats (LTRs). They are often characterized by their pol and gag domains, which are closely related to retroviral proteins. Class II DNA transposons are much less common, and do not require a reverse-transcription step to integrate into the genome. Instead, a transposase, an enzyme that catalyzes transposition, recognizes the terminal inverted repeats (TIRs), excises the TE, and integrates the transposon into the new acceptor site. Both Class I and Class II exhibit complex biological roles in regulation, suppression, and expression [83]. They can evolve to become fully functional genes or duplicate genes to modify regulation [84], [85]. TEs are also known to insert within the sequence of another transposable element, within tandem repeats, or within genes [86]–[88]. In this study, the ratio of Class I to Class II elements (full-length and partial) for *Pinus taeda* was 41∶1. Several full-length DNA transposons were identified with the *de novo* methodology, while none were confirmed via homology-based methods due to the lack of characterized DNA transposons for conifers. DNA transposons represents 0.53% of the full-length repetitive content and 1.52% of the unfiltered repetitive content in our *de novo* analysis. A few angiosperms, such as *Oryza sativa*, are noted to have a much more substantial contribution of DNA transposons relative to retrotransposons [89]; however, most studies have noted that they are at low frequencies when compared with Class I elements [82]. For the angiosperm genomes compared in this study, based on homology, only *Arabidopsis* and cucumber had near equal contributions from both repeat classes (Figure 4A).

Among Class I elements, non-LTR retroelements classified as LINEs were only identified in the *de novo* portion of the analysis and represented 0.71% of the sequence set. LINEs have been found at low frequencies in conifers [90] and vertical transmission has been surmised to be the dominant cause of LINE proliferation in angiosperms [91]. Two ancient LTR superfamilies, Gypsy and Copia, dominate plant genomes and are widespread across chromosomes (consistent with propagation via RNA intermediate) [92]. Though Copia and Gypsy LTR retroelements differ only by the ordering of their RT and INT domains, the ratio between these families varies across plants (Figure 4A). The conifer BACs and fosmids analyzed here (*Pinus taeda, Picea glauca,* and *Taxus mairei*) appear LTR retrotransposon-dense, and DNA transposon and LINE deficient, when compared to the five angiosperms (Figure 4 A). Our evaluation of BAC and fosmid sequences estimated the ratio of Gypsy to Copia in *Pinus taeda* at 1.9∶1, *Picea glauca* at 1∶1.2, and *Taxus mairei* at 1.7∶1 (Table 4; Figure 4A). *Picea glauca,* and *Cucumis sativus* showed slightly greater contributions of Copia over Gypsy elements. *Populus trichocarpa*, however, had a much greater contribution with a Copia to Gypsy ratio of about 3∶1 (Figure 4A). Our full-length analysis was consistent with the homology-based estimates for *Pinus taeda* (Figure 4B), with both yielding a ratio of 2∶1.

Many angiosperm TEs, like the LTR elements of grass genomes, are evolutionarily young and distinguishable [7]. Gymnosperms are markedly different; one hypothesis is that a few elements inserted early, propagated heavily, and diverged via vertical transmission [90]. Phylogenetic analysis based on hybridization studies of 100 RT fragments of Gypsy and Copia elements in 22 conifer species revealed many gymnosperm-specific elements with similar diversity estimates [90]. Support for this hypothesis is reported in the analysis of *Picea glauca* BACs in which transposons appear to have accumulated multiple mutations, indels, and rearrangements [61]. This was again supported in the analysis of *Pinus taeda* BAC sequence, where retroelement frequency distributions support the theory that the genome complexity is largely due to retrotransposon derivatives [3]. In this study, the families identified are numerous, and few annotate to species outside of the *Pinus* genus (Fig. 3B). In addition, we note that only 26% of the genomic sequence sampled is full-length, while 59% is from partial elements. Fourteen (a combination of ten novel and four previously characterized) of the high-copy families constitute only 2.56% of the sequence set, with none exceeding 0.5% individually. The largest novel LTR family, *PtPiedmont*, accounts for only 0.35% of the sequence set (Table 6). Together with diverged elements that are still actively transposing, RT polymerase domains, the most conserved regions of retrotransposons [83], constitute most of our spurious alignments to other genomes.

### Novel high coverage repeats

Among the ten high coverage novel families, six show similarity to known Gypsy elements, while three show similarity to known Copia elements. Nine of the high coverage novel families (*PtConagree, PtPiedmont, PtCumberland, PtOuachita, PtBastrop, PtPineywoods, PtAngelina, PtTalladega, PtOzark*) had no significant (full-length) alignments and are not found in other plant species. The exception, *PtAppalachian*, aligned to several sequences from other plants due to the embedded *PGGYPSYX1*-like sequence. Interestingly, there are virtually no LTR artifacts at either end of the *PGGYPSYX1* insertion within *PtAppalachian*. Observation of the high coverage alignments (Figure 6; Figure S1) shows a substantial amount of LTR degradation in *PtPiedmont*, *PtOuachita*, *PtBastrop*, *PtAppalachian*, *PtPineywoods*, and *PtAngelina*, and each family consensus sequence lacks a detectable PBS, PPT, or both. Many sequences within each cluster are thus nonfunctional and not actively proliferating. Within the high coverage elements, a few have multiple copies of reverse transcriptase, but these copies are usually from different RT lineages. In some cases, the LTR aligns partially *within* the consensus sequence. More specifically, we observed that the region of conservation in the sequence may extend up to 96 bp past the aligned LTR region, as seen in *PtAppalachian*. Embedding of retroelements within other retroelements, especially in flanking regions, has been found in *Beta vulgaris*, where separate Copia and Gypsy elements inserted into an older LTR retrotransposon [93]. This is also seen in *Brassica* species, where nested elements were observed to share similar LTRs [94]. Nesting may have occurred here in *PtOzark*, as well as in *PtPiedmont* and *PtAngelina*. LTR regions delineating the insertion in *PtOzark* can be detected, along with many Pfam alignments within the region of insertion. The fact that internal LTRs in *PtOzark* can be detected seems to conflict with the lack of internal LTRs in *PtAppalachian*. However, the LTRs aligned at only 81% identity, suggesting that significant degradation has already taken place. Furthermore, full-length copies of *PtOzark* are rare, so it may be a recent insertion. *PtPiedmont* and *PtAngelina* both show alignments characteristic of nesting and novel regions unique to each element. These observations, coupled with the fact that about 86% of the sequence set is comprised of low-copy repetitive elements (with individual families accounting for no more than 3% of the sequence set), suggest a complex system of LTR-element retrotransposition. The mechanisms behind the colonization and proliferation of LTRs in gymnosperms are still being elucidated, and remains an interesting topic of research for future studies.

## Supporting Information

Figure S1 **Annotated high copy LTR repeat families.** Multiple alignments of seven high coverage and novel elements were performed using MUSCLE and visualized in Jalview. The final consensus sequence was exported with substitutions resolved, annotated (LTRdigest), and visualized (AnnotationSketch). High coverage elements include *PtPiedmont*, *PtCumberland*, *PtBastrop*, *PtOzark*, *PtAngelina*, *PtConagree*, and *PtTalladega*. These, in addition to *PtOuachita*, *PtAppalachian*, and *PtPineywoods* (Figure 6), represent the 10 high coverage, novel elements.
(TIFF)

Table S1 **Sequence summary for all species.**
(XLSX)

Table S2 **Microsatellite density across all species.**
(XLSX)

Table S3 **Tandem repeat motifs and frequencies in** ***Pinus taeda, Picea glauca,*** **and** ***Taxus mairei.***
(XLSX)

Table S4 **Microsatellite sequence: Pita_MSAT.**
(XLSX)

Table S5 **Partial and full-length repeats in the similarity search against the BAC sequences.**
(XLSX)

Table S6 **REPET seed library composition.** Counts for each classification provided by TEclassifier were aggregated for BAC and fosmid datasets. *No. HSPs* is the number of high scoring hits used to construct the seeds in a particular category. *Length* is the total length in base pairs of all HSPs in a particular category. Per*centage of sequence set* is a function of *Length* and the length of the sequence set. *Percentage of repeats* is the relative ratio of the category of repeats to all repetitive content.
(XLSX)

Table S7 **De novo annotation summary.** Each full-length sequence found in CENSOR was run through our annotation pipeline. Counts for each category were tracked for BAC and fosmid datasets, then combined. *Length* is the sum of all full-length repetitive content in a particular category.
(XLSX)

Table S8 **High coverage families.** Each novel repeat family, aside from the named elements, is represented by one of its members, and each novel repeat element is assigned a unique ID number. *Cumulative length* and *Cumulative percentage of sequence sets* represent the aggregation of the top X highest coverage families, with X denoted by the column *No. families*.
(XLSX)

Table S9 **Selected Pfam profiles.** 147 profiles were queried through keywords such as “retrotransposon”. Selected profiles were used in the annotation of the top ten high coverage elements.
(XLSX)

## References

[1] Bowe LM, Coat G, dePamphilis CW (2000) Phylogeny of seed plants based on all three genomic compartments: Extant gymnosperms are monophyletic and Gnetales' closest relatives are conifers. Proc Natl Acad Sci USA 97: 4092–4097. 10760278 10.1073/pnas.97.8.4092 PMC18159

[2] Peterson DG, Wessler SR, Paterson AH (2002) Efficient capture of unique sequences from eukaryotic genomes. Trends Genet 18: 547–550. 12414178 10.1016/s0168-9525(02)02764-6

[3] Morse A, Peterson D, Islam-Faridi M, Smith K, Magbanua Z, et al (2009) Evolution of genome size and complexity in *Pinus* . PLoS ONE 4: e4332. 19194510 10.1371/journal.pone.0004332 PMC2633040

[4] Ahuja MR, Neale DB (2005) Evolution of genome size in conifers. Silvae Genetica 54: 126–137.

[5] Vitte C, Bennetzen JL (2006) Analysis of retrotransposon structural diversity uncovers properties and propensities in angiosperm genome evolution. Proc Natl Acad Sci USA 103: 17638–17643. 17101966 10.1073/pnas.0605618103 PMC1693799

[6] Neale DB, Kremer A (2011) Forest tree genomics: growing resources and applications. Nat Rev Genet 12: 111–122. 21245829 10.1038/nrg2931

[7] Kovach A, Wegrzyn JL, Parra G, Holt C, Bruening GE, et al (2010) The *Pinus taeda* genome is characterized by diverse and highly diverged repetitive sequences. BMC Genomics 11: 420. 20609256 10.1186/1471-2164-11-420 PMC2996948

[8] Mackay J, Dean JFD, Plomion C, Peterson DG, Canovas FM, et al (2012) Towards decoding the conifer giga-genome. Plant Mol Biol 80: 555–569. 22960864 10.1007/s11103-012-9961-7

[9] Bennett MD, Leitch IJ (2004) Plant DNA C-values database, release 3.0. [online, December 2004]. Available: http://data.kew.org/cvalues/. Accessed 2013 July 22.

[10] McKeand S, Mullin T, Byram T, White T (2003) Deployment of genetically improved loblolly and slash pines in the south. Journal of Forestry 101: 32–37.

[11] Frederick WJ, Lien SJ, Courchene CE, DeMartini NA, Ragauskas AJ, et al (2008) Production of ethanol from carbohydrates from loblolly pine: A technical and economic assessment. Bioresource Technol 99: 5051–5057. 10.1016/j.biortech.2007.08.086 18206369

[12] Mayer KFX, Waugh R, Langridge P, Close TJ, Wise RP, et al (2012) A physical, genetic and functional sequence assembly of the barley genome. Nature 491: 711–716. 23075845 10.1038/nature11543

[13] Wegrzyn JL, Lee JM, Tearse BR, Neale DB (2008) TreeGenes: A forest tree genome database. Int J Plant Genomics 2008: 412875. 18725987 10.1155/2008/412875 PMC2517852

[14] Lorenz WW, Ayyampalayam S, Bordeaux JM, Howe GT, Jermstad KD, et al (2012) Conifer DBMagic: a database housing multiple de novo transcriptome assemblies for 12 diverse conifer species. Tree Genet Genomes 8: 1477–1485.

[15] Shizuya H, Birren B, Kim UJ, Mancino V, Slepak T, et al (1992) Cloning and Stable Maintenance of 300-Kilobase-Pair Fragments of Human DNA in Escherichia-Coli Using an F-Factor-Based Vector. Proc Natl Acad Sci USA 89: 8794–8797. 1528894 10.1073/pnas.89.18.8794 PMC50007

[16] Lander ES, Consortium IHGS, Linton LM, Birren B, Nusbaum C, et al (2001) Initial sequencing and analysis of the human genome. Nature 409: 860–921. 11237011 10.1038/35057062

[17] Rampant PF, Lesur I, Boussardon C, Bitton F, Martin-Magniette ML, et al.. (2011) Analysis of BAC end sequences in oak, a keystone forest tree species, providing insight into the composition of its genome. BMC Genomics 12. 10.1186/1471-2164-12-292 PMC3132169 21645357

[18] Bautista R, Villalobos DP, Diaz-Moreno S, Canton FR, Canovas FM, et al (2007) Toward a Pinus pinaster bacterial artificial chromosome library. Ann Forest Sci 64: 855–864.

[19] Hamberger B, Hall D, Yuen M, Oddy C, Hamberger B, et al (2009) Targeted isolation, sequence assembly and characterization of two white spruce (Picea glauca) BAC clones for terpenoid synthase and cytochrome P450 genes involved in conifer defence reveal insights into a conifer genome. BMC Plant Biol 9: 106. 19656416 10.1186/1471-2229-9-106 PMC2729077

[20] Liu W, Thummasuwan S, Sehgal SK, Chouvarine P, Peterson DG (2011) Characterization of the genome of bald cypress. BMC Genomics 12: 553. 22077969 10.1186/1471-2164-12-553 PMC3228858

[21] Lange C, Holtgrawe D, Schulz B, Weisshaar B, Himmelbauer H (2008) Construction and characterization of a sugar beet (*Beta vulgaris*) fosmid library. Genome 51: 948–951. 18956027 10.1139/G08-071

[22] Hao DC, Ge G, Xiao P, Zhang Y, Yang L (2011) The First Insight into the Tissue Specific *Taxus* Transcriptome via Illumina Second Generation Sequencing. PLoS ONE 6: e21220. 21731678 10.1371/journal.pone.0021220 PMC3120849

[23] Meyer JDF, Deleu W, Garcia-Mas J, Havey MJ (2008) Construction of a fosmid library of cucumber (*Cucumis sativus*) and comparative analyses of the eIF4E and eIF(iso)4E regions from cucumber and melon (*Cucumis melo*). Mol Genet Genomics 279: 473–480. 18273646 10.1007/s00438-008-0326-5

[24] Davis TM, Shields ME, Zhang QA, Tombolato-Terzic D, Bennetzen JL, et al.. (2010) An examination of targeted gene neighborhoods in strawberry. BMC Plant Biol 10. 10.1186/1471-2229-10-81 PMC2890015 20441596

[25] Kim UJ, Shizuya H, Dejong PJ, Birren B, Simon MI (1992) Stable Propagation of Cosmid Sized Human DNA Inserts in an F-Factor Based Vector. Nucleic Acids Res 20: 1083–1085. 1549470 10.1093/nar/20.5.1083 PMC312094

[26] Kumar A, Bennetzen JL (1999) Plant retrotransposons. Annu Rev of Genet 33: 479–532. 10690416 10.1146/annurev.genet.33.1.479

[27] Bennetzen JL (2005) Transposable elements, gene creation and genome rearrangement in flowering plants. Curr Opin Genet Dev 15: 621–627. 16219458 10.1016/j.gde.2005.09.010

[28] Kaul S, Koo HL, Jenkins J, Rizzo M, Rooney T, et al (2000) Analysis of the genome sequence of the flowering plant *Arabidopsis thaliana* . Nature 408: 796–815. 11130711 10.1038/35048692

[29] Wicker T, Zimmermann W, Perovic D, Paterson AH, Ganal M, et al (2005) A detailed look at 7 million years of genome evolution in a 439 kb contiguous sequence at the barley Hv-eIF4E locus: recombination, rearrangements and repeats. Plant J 41: 184–194. 15634196 10.1111/j.1365-313X.2004.02285.x

[30] Jaillon O, Aury JM, Noel B, Policriti A, Clepet C, et al (2007) The grapevine genome sequence suggests ancestral hexaploidization in major angiosperm phyla. Nature 449: 463–U465. 17721507 10.1038/nature06148

[31] Piegu B, Guyot R, Picault N, Roulin A, Saniyal A, et al (2006) Doubling genome size without polyploidization: Dynamics of retrotransposition-driven genomic expansions in *Oryza australiensis*, a wild relative of rice. Genome Res 16: 1262–1269. 16963705 10.1101/gr.5290206 PMC1581435

[32] Burleigh JG, Barbazuk WB, Davis JM, Morse AM, Soltis PS (2012) Exploring Diversification and Genome Size Evolution in Extant Gymnosperms through Phylogenetic Synthesis. Journal of Botany 2012, Article ID 292857: 6 pages.

[33] Hao D, Yang L, Xiao P (2011) The first insight into the *Taxus* genome via fosmid library construction and end sequencing. Mol Genet Genomics 285: 197–205. 21207064 10.1007/s00438-010-0598-4

[34] L'Homme Y, Seguin A, Tremblay F (2000) Different classes of retrotransposons in coniferous spruce species. Genome 43: 1084–1089. 11195342

[35] Magbanua ZV, Ozkan S, Bartlett BD, Chouvarine P, Saski CA, et al (2011) Adventures in the enormous: a 1.8 million clone BAC library for the 21.7 Gb genome of loblolly pine. PLoS ONE 6: e16214. 21283709 10.1371/journal.pone.0016214 PMC3025025

[36] Peterson D, Tomkins J, Frisch D, Wing R, Paterson A ( 2000) Construction of plant bacterial artificial chromosome (BAC) libraries: an illustrated guide. Journal of Agricultural Genomics 5: 1–100.

[37] Aronesty E (2011) ea-utils: Command-line tools for processing biological sequencing data. Available: https://code.google.com/p/ea-utils/. Accessed 2013 July 22.

[38] Li H, Durbin R (2010) Fast and accurate long-read alignment with Burrows-Wheeler transform. Bioinformatics 26: 589–595. 20080505 10.1093/bioinformatics/btp698 PMC2828108

[39] Li RQ, Zhu HM, Ruan J, Qian WB, Fang XD, et al (2010) De novo assembly of human genomes with massively parallel short read sequencing. Genome Res 20: 265–272. 20019144 10.1101/gr.097261.109 PMC2813482

[40] Benson G (1999) Tandem repeats finder: a program to analyze DNA sequences. Nucleic Acids Res 27: 573–580. 9862982 10.1093/nar/27.2.573 PMC148217

[41] Goodstein DM, Shu SQ, Howson R, Neupane R, Hayes RD, et al (2012) Phytozome: a comparative platform for green plant genomics. Nucleic Acids Res 40: D1178–D1186. 22110026 10.1093/nar/gkr944 PMC3245001

[42] Kohany O, Gentles AJ, Hankus L, Jurka J (2006) Annotation, submission and screening of repetitive elements in Repbase: RepbaseSubmitter and Censor. BMC Bioinformatics 7: 474. 17064419 10.1186/1471-2105-7-474 PMC1634758

[43] Kamm A, Doudrick RL, HeslopHarrison JS, Schmidt T (1996) The genomic and physical organization of Ty1-copia-like sequences as a component of large genomes in *Pinus elliottii* var *elliottii* and other gymnosperms. Proc Natl Acad Sci USA 93: 2708–2713. 8610105 10.1073/pnas.93.7.2708 PMC39695

[44] Rocheta M, Cordeiro J, Oliveira M, Miguel C (2007) PpRT1: the first complete gypsy-like retrotransposon isolated in *Pinus pinaster* . Planta 225: 551–562. 17008993 10.1007/s00425-006-0370-5

[45] Rocheta M, Carvalho L, Viegas W, Morais-Cecilio L (2012) Corky, a gypsy-like retrotransposon is differentially transcribed in *Quercus suber* tissues. BMC Res Notes 5: 432. 22888907 10.1186/1756-0500-5-432 PMC3465219

[46] Wicker T, Sabot F, Hua-Van A, Bennetzen JL, Capy P, et al (2007) A unified classification system for eukaryotic transposable elements. Nat Rev Genet 8: 973–982. 17984973 10.1038/nrg2165

[47] Flutre T, Duprat E, Feuillet C, Quesneville H (2011) Considering Transposable Element Diversification in De Novo Annotation Approaches. PLoS ONE 6(1): e16526. 21304975 10.1371/journal.pone.0016526 PMC3031573

[48] Quesneville H, Nouaud D, Anxolabehere D (2003) Detection of new transposable element families in *Drosophila melanogaster* and *Anopheles gambiae* genomes. J Mol Evol 57: S50–S59. 15008403 10.1007/s00239-003-0007-2

[49] Bao ZR, Eddy SR (2002) Automated de novo identification of repeat sequence families in sequenced genomes. Genome Res 12: 1269–1276. 12176934 10.1101/gr.88502 PMC186642

[50] Edgar RC, Myers EW (2005) PILER: identification and classification of genomic repeats. Bioinformatics 21: I152–I158. 15961452 10.1093/bioinformatics/bti1003

[51] Huang XQ (1994) On Global Sequence Alignment. Comput Appl Biosci 10: 227–235. 7922677 10.1093/bioinformatics/10.3.227

[52] Ellinghaus D, Kurtz S, Willhoeft U (2008) LTRharvest, an efficient and flexible software for de novo detection of LTR retrotransposons. BMC Bioinformatics 9: 18. 18194517 10.1186/1471-2105-9-18 PMC2253517

[53] Dondoshansky I ( 2002) Blastclust (NCBI Software Development Toolkit). 61 edition NCBI, Bethesda, MD.

[54] Li XG, Wu HX, Southerton SG (2011) Transcriptome profiling of wood maturation in *Pinus radiata* identifies differentially expressed genes with implications in juvenile and mature wood variation. Gene 487: 62–71. 21839815 10.1016/j.gene.2011.07.028

[55] Edgar RC (2010) Search and clustering orders of magnitude faster than BLAST. Bioinformatics 26: 2460–2461. 20709691 10.1093/bioinformatics/btq461

[56] Edgar RC (2004) MUSCLE: a multiple sequence alignment method with reduced time and space complexity. BMC Bioinformatics 5: 1–19. 15318951 10.1186/1471-2105-5-113 PMC517706

[57] Waterhouse AM, Procter JB, Martin DMA, Clamp M, Barton GJ (2009) Jalview Version 2-a multiple sequence alignment editor and analysis workbench. Bioinformatics 25: 1189–1191. 19151095 10.1093/bioinformatics/btp033 PMC2672624

[58] Steinbiss S, Willhoeft U, Gremme G, Kurtz S (2009) Fine-grained annotation and classification of de novo predicted LTR retrotransposons. Nucleic Acids Res 37: 7002–7013. 19786494 10.1093/nar/gkp759 PMC2790888

[59] Finn RD, Mistry J, Tate J, Coggill P, Heger A, et al (2010) The Pfam protein families database. Nucleic Acids Res 38: D211–D222. 19920124 10.1093/nar/gkp985 PMC2808889

[60] Tuskan G, Difazio S, Jansson S, Bohlmann J, Grigoriev I, et al (2006) The genome of black cottonwood, *Populus trichocarpa* (Torr. & Gray). Science 313: 1596–1604. 16973872 10.1126/science.1128691

[61] Hamberger B, Hall D, Yuen M, Oddy C, Hamberger B, et al (2009) Targeted isolation, sequence assembly and characterization of two white spruce (*Picea glauca*) BAC clones for terpenoid synthase and cytochrome P450 genes involved in conifer defence reveal insights into a conifer genome. BMC Plant Biol 9: 106. 19656416 10.1186/1471-2229-9-106 PMC2729077

[62] Parks M, Cronn R, Liston A (2009) Increasing phylogenetic resolution at low taxonomic levels using massively parallel sequencing of chloroplast genomes. BMC Biol 7: 84. 19954512 10.1186/1741-7007-7-84 PMC2793254

[63] Stanke M, Keller O, Gunduz I, Hayes A, Waack S, et al (2006) AUGUSTUS: ab initio prediction of alternative transcripts. Nucleic Acids Research 34: W435–W439. 16845043 10.1093/nar/gkl200 PMC1538822

[64] Holt C, Yandell M (2011) MAKER2: an annotation pipeline and genome-database management tool for second-generation genome projects. BMC Bioinformatics 12. 10.1186/1471-2105-12-491 PMC3280279 22192575

[65] Parra G, Bradnam K, Korf I (2007) CEGMA: a pipeline to accurately annotate core genes in eukaryotic genornes. Bioinformatics 23: 1061–1067. 17332020 10.1093/bioinformatics/btm071

[66] Insititute for Systems Biology: RepeatMasker. Avalaible: http://www.repeatmasker.org/. Accessed 2013 July 22.

[67] Wu J, Gu YQ, Hu Y, You FM, Dandekar AM, et al (2012) Characterizing the walnut genome through analyses of BAC end sequences. Plant Mol Biol 78: 95–107. 22101470 10.1007/s11103-011-9849-y

[68] Ming R, Hou SB, Feng Y, Yu QY, Dionne-Laporte A, et al (2008) The draft genome of the transgenic tropical fruit tree papaya (*Carica papaya* Linnaeus). Nature 452: 991–U997. 18432245 10.1038/nature06856 PMC2836516

[69] Richards EJ, Ausubel FM (1988) Isolation of a Higher Eukaryotic Telomere from *Arabidopsis Thaliana* . Cell 53: 127–136. 3349525 10.1016/0092-8674(88)90494-1

[70] Kossack DS, Kinlaw CS (1999) IFG, a gypsy-like retrotransposon in *Pinus* (Pinaceae), has an extensive history in pines. Plant Mol Biol 39: 417–426. 10092171 10.1023/a:1006115732620

[71] Jeffreys AJ, Neil DL, Neumann R (1998) Repeat instability at human minisatellites arising from meiotic recombination. Embo Journal 17: 4147–4157. 9670029 10.1093/emboj/17.14.4147 PMC1170747

[72] Richard GF, Kerrest A, Dujon B (2008) Comparative genomics and molecular dynamics of DNA repeats in eukaryotes. Microbiol Mol Biol Rev 72: 686–727. 19052325 10.1128/MMBR.00011-08 PMC2593564

[73] Gemayel R, Vinces MD, Legendre M, Verstrepen KJ (2010) Variable tandem repeats accelerate evolution of coding and regulatory sequences. Annu Rev Genet 44: 445–477. 20809801 10.1146/annurev-genet-072610-155046

[74] Treangen TJ, Salzberg SL (2012) Repetitive DNA and next-generation sequencing: computational challenges and solutions. Nature Rev Genet 13: 36–46. 10.1038/nrg3117 PMC3324860 22124482

[75] Li YC, Korol AB, Fahima T, Beiles A, Nevo E (2002) Microsatellites: genomic distribution, putative functions and mutational mechanisms: a review. Mol Ecol 11: 2453–2465. 12453231 10.1046/j.1365-294x.2002.01643.x

[76] Schmidt A, Doudrick RL, Heslop-Harrison JS, Schmidt T (2000) The contribution of short repeats of low sequence complexity to large conifer genomes. Theor Appl Genet 101: 7–14.

[77] Cavagnaro PF, Senalik DA, Yang L, Simon PW, Harkins TT, et al (2010) Genome-wide characterization of simple sequence repeats in cucumber (*Cucumis sativus* L.). BMC Genomics 11: 569. 20950470 10.1186/1471-2164-11-569 PMC3091718

[78] Nagarajan N, Navajas-Pérez R, Pop M, Alam M, Ming R, et al (2008) Genome-Wide Analysis of Repetitive Elements in Papaya. Trop Plant Biol 1: 191–201.

[79] Smith DN, Devey ME (1994) Occurrence and inheritance of microsatellites in *Pinus radiata* . Genome 37: 977–983. 7828844 10.1139/g94-138

[80] Elsik CG, Williams CG (2001) Families of clustered microsatellites in a conifer genome. Mol Genet Genomics 265: 535–542. 11405637 10.1007/s004380100443

[81] Schnable PS, Ware D, Fulton RS, Stein JC, Wei FS, et al (2009) The B73 Maize Genome: Complexity, Diversity, and Dynamics. Science 326: 1112–1115. 19965430 10.1126/science.1178534

[82] Civáň P, Švec M, Hauptvogel P (2011) On the Coevolution of Transposable Elements and Plant Genomes. Journal of Botany 2011, Article ID 893546, 9 pages.

[83] Slotkin RK, Martienssen R (2007) Transposable elements and the epigenetic regulation of the genome. Nat Rev Genet 8: 272–285. 17363976 10.1038/nrg2072

[84] Jurka J, Kapitonov VV, Kohany O, Jurka MV (2007) Repetitive sequences in complex genomes: structure and evolution. Annu Rev Genomics Hum Genet 8: 241–259. 17506661 10.1146/annurev.genom.8.080706.092416

[85] Flagel LE, Wendel JF (2009) Gene duplication and evolutionary novelty in plants. New Phytol 183: 557–564. 19555435 10.1111/j.1469-8137.2009.02923.x

[86] Kumekawa N, Ohmido N, Fukui K, Ohtsubo E, Ohtsubo H (2001) A new gypsy-type retrotransposon, RIRE7: preferential insertion into the tandem repeat sequence TrsD in pericentromeric heterochromatin regions of rice chromosomes. Mol Genet Genomics 265: 480–488. 11405631 10.1007/s004380000436

[87] Jiang N, Wessler SR (2001) Insertion preference of maize and rice miniature inverted repeat transposable elements as revealed by the analysis of nested elements. Plant Cell 13: 2553–2564. 11701888 10.1105/tpc.010235 PMC139471

[88] Miyao A, Tanaka K, Murata K, Sawaki H, Takeda S, et al (2003) Target site specificity of the Tos17 retrotransposon shows a preference for insertion within genes and against insertion in retrotransposon-rich regions of the genome. Plant Cell 15: 1771–1780. 12897251 10.1105/tpc.012559 PMC167168

[89] Feschotte C, Pritham EJ (2007) DNA transposons and the evolution of eukaryotic genomes. Annu Rev of Genet 41: 331–368. 18076328 10.1146/annurev.genet.40.110405.090448 PMC2167627

[90] Friesen N, Brandes A, Heslop-Harrison JS (2001) Diversity, origin, and distribution of retrotransposons (gypsy and copia) in conifers. Mol Biol Evol 18: 1176–1188. 11420359 10.1093/oxfordjournals.molbev.a003905

[91] Noma K, Ohtsubo E, Ohtsubo H (1999) Non-LTR retrotransposons (LINEs) as ubiquitous components of plant genomes. Molecular and General Genetics 261: 71–79. 10071212 10.1007/s004380050943

[92] Kejnovsky E, Hawkins J, Feschotte C (2012) Plant Transposable Elements: Biology and Evolution. In: Wendel JF, Greilhuber J, Dolezel J, Leitch IJ, editors. Plant Genome Diversity Volume 1: Springer Vienna. 17–34.

[93] Kuykendall D, Shao J, Trimmer K (2009) A nest of LTR retrotransposons adjacent the disease resistance-priming gene NPR1 in *Beta vulgaris* L. U.S. hybrid H20. Int J Plant Genomics 2009, Article ID 576742: 8 pages. 10.1155/2009/576742 PMC2669250 19390694

[94] Wei L, Xiao M, An Z, Ma B, S. Mason A, et al (2012) New insights into nested long terminal repeat retrotransposons in *Brassica* species. Mol Plant 6: 470–482. 22930733 10.1093/mp/sss081

