# Supplementary figures and images for: Insights into the Loblolly Pine Genome: Characterization of BAC and Fosmid Sequences
Source: PLoS One. 2013 Sep 4;8(9):e72439. doi: 10.1371/journal.pone.0072439 (PMC3762812; doi:10.1371/journal.pone.0072439)

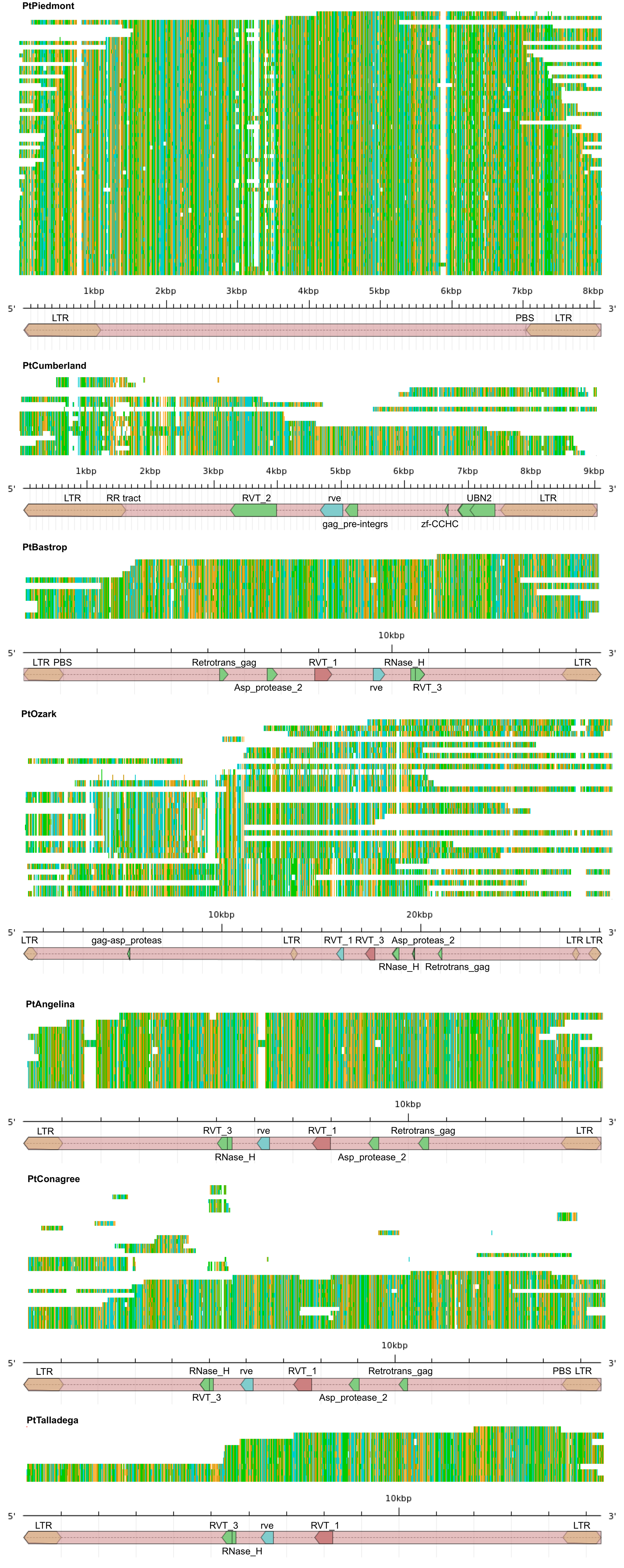

Supplement: Figure S1 — Annotated high copy LTR repeat families. Multiple alignments of seven high coverage and novel elements were performed using MUSCLE and visualized in Jalview. The final consensus sequence was exported with substitutions resolved, annotated (LTRdigest), and visualized (AnnotationSketch). High coverage elements include PtPiedmont, PtCumberland, PtBastrop, PtOzark, PtAngelina, PtConagree, and PtTalladega. These, in addition to PtOuachita, PtAppalachian, and PtPineywoods (Figure 6), represent the 10 high coverage, novel elements. (TIFF) [file pone.0072439.s001.tiff]
